# Enhanced decision technique for optimized crude oil pretreatment under disc spherical fuzzy Aczel Alsina aggregation information

**DOI:** 10.1038/s41598-024-62036-9

**Published:** 2024-07-02

**Authors:** Qazi Adnan Ahmad, Shahzaib Ashraf, Wania Iqbal, Ma Li Qiang

**Affiliations:** 1 School of Mines, China University of Mining and Technology, Xuzhou, Jiangsu China; 2 Institute of Mathematics, Khwaja Fareed University of Engineering & Information Technology, Rahim Yar Khan, 64200 Pakistan; 3 College of Energy and Mining Engineering, Shandong University of Science and Technology, Qingdao, 266590 China; 4 Key Laboratory of Xinjiang Coal Resources Green Mining (Xinjiang Institute of Engineering), Ministry of Education, Ürümqi, 830023 China

**Keywords:** Crude oil treatment, Disc spherical fuzzy set (D-SFS), MARCOS method, MEREC method, SWARA method, Applied mathematics, Computational science, Information technology, Scientific data

## Abstract

Crude oil, the backbone of modern industry, holds unparalleled significance as a global energy cornerstone. Unlocking its potential hinges on effective pretreatment techniques, ensuring purity, and maximizing efficiency. This study extends the established Spherical Fuzzy Set paradigm to explore the domain of Disc Spherical Fuzzy Sets (D-SFSs) in critical decision-making for crude oil preparation. Investigating D-SFSs within the Aczel Alsina norm, the research employs comparison rules, conversion rules, and distance metrics. Primary operations of the Aczel Alsina norm in D-SFSs are examined, laying the groundwork for introducing unique aggregation operations within this framework. The paper’s primary aim is to propose a hybrid method, termed MEREC-SWARA-MARCOS-D-SFSs Multiple Attribute Group Decision Making, which integrates the aforementioned aggregation procedures. A case study on crude oil pretreatment validates the effectiveness of the proposed method. Furthermore, a comprehensive comparison with CoCoSo underscores the reliability of the method. This study represents a significant stride in enhancing decision-making by providing a robust framework to tackle complex situations, particularly in the critical domain of crude oil pretreatment.

## Introduction

In the oil and gas sector, improving the pretreatment of crude oil is a crucial frontier that aims to maximize the extraction and refining operations. Modern technologies in this field greatly enhance the effectiveness of separation and provide refined goods of superior quality. Industry sources state that the use of cutting-edge pretreatment techniques has improved separation efficiency significantly, lowering water content by 95% and contaminants by up to 90%, significantly improving the quality of the recovered crude oil. The integration of advanced membrane filtration and electrostatic coalescers has resulted in a noteworthy drop in energy consumption. The statistics indicate a noteworthy 20% reduction in overall energy needs. Furthermore, these developments comply with strict environmental regulations, which leads to a significant reduction in carbon emissions and trash production in the pretreatment stage. These systems are scalable, as demonstrated by their capacity to manage different amounts of crude oil, demonstrating flexibility in response to variations in output. These developments highlight a revolutionary move toward more effective, eco-friendly, and commercially feasible crude oil pretreatment methods as the industry continues to emphasize sustainable practices.

The oil and gas industry’s decision-making (DM) processes are directly and significantly impacted by developments in crude oil pretreatment technologies. Pretreatment using state-of-the-art technology improves the DM environment for refiners and oil producers. For example, decision-makers have to balance the initial investment costs with the advantages of improved separation efficiency when deciding which pretreatment techniques to use. As the industry sees a quantifiable decrease in contaminants and an improvement in water content, which directly affects the quality of the refined goods, data-driven DM becomes essential. The energy consumption of sophisticated pretreatment methods, such as membrane filtration and electrostatic coalescers, must be carefully considered before selecting one over the other. Figures showing the decline in energy demand may be analyzed by decision-makers to help them meet cost-saving goals and sustainability goals.

Furthermore, while making decisions, consideration must be given to how pretreatment technologies may affect the environment. Organizations all around the world are emphasizing environmentally friendly practices; therefore, decision-makers need to assess how implementing sophisticated pretreatment systems can lower carbon emissions and waste production. Decision-making is further aided by these systems’ scalability and compatibility, which enable smooth integration into the current infrastructure and flexibility in managing fluctuating amounts of crude oil.

### Literature survey

Scholars have shown significant interest in oil treatment research. Oil emulsification was examined by Thompson et al. (1985)^1^, ultrasound applications by Ye et al.^2^, and ultrasonic-electric desalting by Guoxiang et al.^3^. Hu et al.^4^ offered a review on the development of bio-crude oil, whereas Khajehesamedini et al.^5^ addressed both theoretical and experimental issues. Additional research includes the work of Babalola et al.^6^ on the pretreatment of heavy crude oil, Hajeeh’s^7^ analysis of the performance of water desalination plants in fuzzy DM, and Fetana’s and Tayebi’s^8^ investigation of petroleum refinery effluents utilizing picture fuzzy techniques. Studies such as^9^ and^10^ demonstrate how fuzzy DM has been used by a variety of researchers in various areas of oil treatment. Together, these pieces demonstrate the variety of methods for improving oil treatment procedures.

These studies improve our knowledge of the topic by offering insightful information on the intricacies of pretreatment through the use of fuzzy logic and DM approaches. As^11^ and^12^ have shown, fuzzy sets (FSs) provide a more complex picture of membership that takes into account different levels of affiliation. This idea is expanded upon by Intuitionistic FSs (IFSs)^13^, which provide non-membership degrees for imprecise data. Circular IFS (C-IFS) are a powerful tool for representing ambiguity in grades of belonging and non-belonging, as introduced by Atanassov^14^. For C-IFSs, certain distance metrics^15^ enhance flexibility, particularly in cases where a circular format is favored. By examining techniques like TOPSIS^16^, TODIM^17^, and the expanded EDAS approach^18^, these studies advance our capacity to solve the particular issues in this domain and contribute to DM methodologies in oil pretreatment.

Yager extended the use of IFSs by introducing the Pythagorean Fuzzy Set (PyFS)^19^. Comprehensive investigations and applications described in^20^ and^21^ have bolstered the substantial interest in this addition. Similar to how IFS developed into C-IFS^22,23^, PyFS has led to the creation of disc PyFSs and circular PyFSs. Olgun and Ünver^24^ and Alsattar et al.^25^ have extended MADM and 3-way decision making in this area. This development highlights how fuzzy set frameworks are adaptable to different representation strategies designed for different types of DM situations.

The notion of spherical FSs (SFSs) was expanded upon by Ashraf and his fellow’s work^26^, which took abstinence, non-belonging degree, and belonging degree into consideration. Due to its closer alignment with human nature, this structure is currently the subject of intense research. In his study^27^, Ashraf and Abdullah made more contributions to the subject by investigating the applications of SFS in Multiple Attribute Decision Making (MADM). Rafiq et al.^28^ and Khan et al.^29^ explore similarity and distance in SFSs and offer important new insights into this developing topic with their concepts of cosine similarity. By expanding SFSs to Disc SFSs (D-SFS), Ashraf et al.^30^ achieved a noteworthy breakthrough. By adding circular components to the dimensions of abstention, non-belonging, and belonging, this addition improves the framework’s adaptability and representational power. In C-SFS^31^, sugeno weber based aggregation operations are introduced. This invention adds to a more thorough strategy, which is especially helpful in a variety of DM situations.

The MADM technique is a powerful way of tackling a wide range of problems, assessing options, and assisting users with problem mapping. The choice of criteria weights is critical in the decision-making process since choosing proper criterion weights is required for successfully ranking alternatives. However, criteria weight information in MADM situations is occasionally unknown, necessitating the development of algorithms for determining these weights. There are several approaches available in the literature for this purpose. The MEREC methodology, introduced by Keshavarz et al.^32^, stands out as a reliable way for determining objective criteria weights. Several studies have used the MEREC-CoCoSo multi-criteria technique, including the work of Marinkovic et al.^33^, who used it to evaluate the use of waste and recycled materials in production. Mishra et al.^34^ connected MEREC with MOORA, Wan et al.^35^ integrated it with CoCoSo, and Gao et al.^36^ used it with SFS data. Furthermore, the SWARA methodology, developed by Kersuliene et al.^37^, has been identified as an efficient way for generating subjective criteria weights (SCWs). Furthermore, many weight approaches, as stated by Chen^38^, have been investigated. SWARA, paired with MARCOS has been used in a variety of investigations^39^, including those conducted by Chaurasiya and Jain^40^ and others. Some integrated approaches have been presented to handle MCDM difficulties, such as Rani et al.’s SWARA-ARAS method for HCWT^41,42^. The MARCOS approach, first established by Stevic et al.^43^, has been utilized in traffic analysis and project management software evaluation. Its variation GREY-MARCOS is utilized in supplier selection^44^, respectively. Several academics have expanded the standard MARCOS approach to accept hazy contexts, with applications in a variety of fields, as indicated by the works of Stankovic et al.^45^, Puska et al.^46^ Chaurasiya and Jain^47^ and Kumar et al.^48^.

### Motivation and contributions

The basic objective of this research is to create an integrated decision-making technique, D-SF-MEREC-SWARA-MARCOS, that is especially designed to effectively deal with the inherent ambiguity and uncertainty in evaluations produced by decision experts. Initially, the focus is on computing objective weights using the MEREC approach, followed by assessing subjective weights using the SWARA method. Then, the combined criteria weights are generated, and the MARCOS approach is utilized to compute alternative ranks. The primary benefit of using the Disc SF MARCOS technique is that it incorporates D-SF context points, including D-SF-ideal, anti-ideal, and abstinence solutions, along with radius, during the model creation process. Furthermore, this technique introduces utility functions, as well as aggregation and measurement functions. It is anticipated that this novel approach will be particularly beneficial for handling a diverse array of criteria and alternatives.

The article covers the following major points:

1. Development of Aczel–Alsina-based Aggregation Operators in the D-SF Framework. This study is the first to integrate Azcel-Alsina-based aggregation operators into the D-SF framework, offering a unique decision-making technique.
2. Development of an integrated MCDM model, namely MEREC-SWARA-MARCOS, within a D-SFS environment. This model includes the computation of decision experts’ weights in D-SFS and an aggregate of D-SFNs.
3. Objective criterion weights are calculated using the MEREC technique, whereas subjective criterion weights are calculated using SWARA. Next, the combined criteria weights are determined.
4. An empirical case study on crude oil pretreatment is discussed. The study’s goal is to find the optimum treatment strategy to demonstrate the potential and effectiveness of the proposed MEREC-SWARA-MARCOS method in the D-SFS setting.
5. Conducting a comparison examination of the proposed approach against current techniques to ensure its efficacy and logic. The purpose of this comparison is to evaluate the validity and performance of the well-established D-SF-MEREC-SWARA-MARCOS methodology.

#### Practical application

The practical application of disc SFSs (D-SFS) in crude oil pretreatment represents a significant advancement in the oil industry. A flexible framework for addressing the intricacies and inherent uncertainties in the crude oil treatment process is offered by D-SFS. This novel method takes into account characteristics like impurity, viscosity, and chemical compositions to provide a more detailed picture of the elements that influence decisions. The optimization of crude oil pretreatment can result in enhanced efficiency in impurity separation, desalting, and dewatering by employing D-SFS in the DM process. D-SFS is a useful tool for improving the overall dependability and efficiency of the pretreatment stage in the oil production workflow because of its flexibility in responding to the unique requirements of treating crude oil.

#### Organization of the study

The layout of the paper is as follows: A survey of the literature is included in the introductory in “Introduction” section to lay the groundwork for the investigation. Essential ideas forming the information that follows are presented in “Disc spherical fuzzy sets” section. In “Exploring Azcel–Alsina operations in D-SFSs” section, the fundamental operations of the Aczel Alsina norm are examined, with characteristics supported by proofs. The Aczel–Alsina norm for D-SFSs aggregating processes and the characteristics they possess are introduced in “Aggregation Operators for D-SFS” section. A new MADM method is presented in “D-SF decision matrix construction” section. In “Case Study: Advancements in Crude Oil Pretreatment Systems” section, a case study with a solved numerical example is presented. The comparative analysis is carried out in Section 7. Section 8 concludes the article by providing a summary of its main conclusions.

## Preliminaries

In this section, we’ll explore pivotal topics crucial to crafting this article, delving into their significance and impact.

### Definition 2.1

^14^

Assuming $\intercal$ is the universal set and $\Delta$ is a subset of $\intercal$, the C-IFS is defined as follows.

1 $$\begin{aligned} \Delta =\{ (\epsilon ,\xi _{\Delta }(\epsilon ), \chi _{\Delta }(\epsilon );\curlyvee _{\Delta } )|\epsilon \in \intercal \} \end{aligned}$$

where $\xi _{\Delta } and \chi _{\Delta }:\intercal \rightarrow [0,1], \chi _{\Delta }(\epsilon ):\intercal \rightarrow [0,1]$ indicating positive grade, negative grade with $0\le \xi _{\Delta }(\epsilon )+\chi _{\Delta }(\epsilon )\le 1$ Each element in the C-IFS is represented by a circle with a center at $(\xi _{\Delta }(\epsilon ), \chi _{\Delta }(\epsilon ))$ and radius $\curlyvee _{\Delta } \in [0,1]$. This is in contrast to the typical IFS, where elements are denoted by points within the intuitionistic fuzzy interpretation triangle.

### Definition 2.2

^22^

Imagine $\intercal$ be the universal set and $r\in [0,1]$. The C-PFS in $\intercal$ is given by

$$\begin{aligned} \Delta =\{ (\epsilon ,\xi _{\Delta }(\epsilon ),\chi _{\Delta }(\epsilon );r)|\epsilon \in \intercal \} \end{aligned}$$

frequently described as a pythagorean fuzzy set, with $\xi _{\Delta }(\epsilon ): \intercal \rightarrow [0,1]$ and $\chi _{\Delta }(\epsilon ): \intercal \rightarrow [0,1]$ representing positive and negative grade, respectively, while adhering to the specified condition.

$$\begin{aligned} 0\le (\xi _{\Delta }(\epsilon ))^{2}+(\chi _{\Delta }(\epsilon ))^{2} \le 1 \end{aligned}$$

The coordinates $(\xi _{\Delta }(\epsilon ), \chi _{\Delta }(\epsilon ))$ on the plane correspond to the radius of the circle, denoted as $\curlyvee$.

### Definition 2.3

^23^

For $\curlyvee (\epsilon ) \in [0,1]$ a D-PFS in $\intercal$ is given by:

$$\begin{aligned} \Delta =\{ (\epsilon ,\xi _{\Delta }(\epsilon ),\chi _{\Delta }(\epsilon );\curlyvee (\epsilon ))|\epsilon \in \intercal \} \end{aligned}$$

where $\xi _{\Delta }$ and $\chi _{\Delta }$ are functions mapping from the universal set $\intercal$ to the closed interval [0,1] with

$$\begin{aligned} 0\le (\xi _{\Delta }(\epsilon ))^{2}+(\chi _{\Delta }(\epsilon ))^{2} \le 1 (\forall \epsilon \in \intercal ) \end{aligned}$$

The coordinates $(\xi _{\Delta }(\epsilon ), \chi _{\Delta }(\epsilon ))$ on the plane correspond to the radius of the circle, denoted as $\curlyvee$. The radius of the circle, denoted by$\curlyvee$, is defined by the coordinates $(\xi _{\Delta }(\epsilon ), \chi _{\Delta }(\epsilon ))$ on the plane. This circle illustrates the grades of positive and negative of $\epsilon \in \intercal .$

### Disc spherical fuzzy sets

#### Definition 2.4

^30^

A C-SFS in $\intercal$ is given by:

2 $$\begin{aligned} \Delta =\big \{ (\epsilon ,\xi _{\Delta }(\epsilon ), \eta _{\Delta }(\epsilon ),\chi _{\Delta }(\epsilon );\curlyvee )|\epsilon \in \intercal \big \} \end{aligned}$$

will be characterized as a C-SFS in which $\xi _{\Delta }, \eta _{\Delta }$ and $\chi _{\Delta }$ are functions mapping from the universal set $\intercal$ to the closed interval [0,1], essentially represent positive, neutral, and negative grade. Additionally, $\xi _{\Delta }, \eta _{\Delta }$ and $\chi _{\Delta }$ adhere to the specified criteria:

3 $$\begin{aligned} 0\le (\xi _{\Delta }(\epsilon ))^{2}+ (\eta _{\Delta }(\epsilon ))^{2}+(\chi _{\Delta }(\epsilon ))^{2} \le 1. (\forall \epsilon \in \intercal ) \end{aligned}$$

The radius of the circle around it, denoted as $\curlyvee$, is determined by the point $\left( \xi _{\Delta }(\epsilon ), \eta _{\Delta }(\epsilon ) \chi _{\Delta }(\epsilon )\right)$ on the sphere. This circle represents the degrees of positivity, neutrality, and negativity of $\epsilon$ within $\intercal$.

In this C-SFS, each element is represented by a circle with a center $\left( \xi _{\Delta }(\epsilon ), \eta _{\Delta }(\epsilon ) \chi _{\Delta }(\epsilon )\right)$ at and a radius $\curlyvee$, in contrast to a point in the spherical fuzzy interpretation triangle as seen in typical SFSs. This novel set is an advancement beyond the standard SFS, as denoted by $\Delta = \Delta _o = \{\langle \epsilon , \xi _{\Delta }(\epsilon ), \eta _{\Delta }(\epsilon ), \chi _{\Delta }(\epsilon ); 0 \rangle \}$. However, the C-SFS with $\curlyvee > 0$ does not conform to a normal SFS.

#### Definition 2.5

^30^

A D-SFS in $\intercal$ is given by:

4 $$\begin{aligned} \Delta =\big \{ (\epsilon ,\xi _{\Delta }(\epsilon ), \eta _{\Delta }(\epsilon ),\chi _{\Delta }(\epsilon );\curlyvee (\epsilon ))|\epsilon \in \intercal \big \} \end{aligned}$$

will be characterized as a D-SFS in which $\xi _{\Delta }, \eta _{\Delta }$ and $\chi _{\Delta }$ are functions mapping from the universal set $\intercal$ to the closed interval [0,1], essentially represent positive, neutral, and negative grade. Additionally, $\xi _{\Delta }, \eta _{\Delta }$ and $\chi _{\Delta }$ adhere to the specified criteria:

5 $$\begin{aligned} 0\le (\xi _{\Delta }(\epsilon ))^{2}+ (\eta _{\Delta }(\epsilon ))^{2}+(\chi _{\Delta }(\epsilon ))^{2} \le 1. (\forall \epsilon \in \intercal ) \end{aligned}$$

The radius of the circle around it, denoted as $\curlyvee (\epsilon )$, is determined by the point $\left( \xi _{\Delta }(\epsilon ), \eta _{\Delta }(\epsilon ) \chi _{\Delta }(\epsilon )\right)$ on the sphere. This circle represents the degrees of positivity, neutrality, and negativity of $\epsilon$ within $\intercal$.

In this D-SFS, each element is represented by a circle with a center $\left( \xi _{\Delta }(\epsilon ), \eta _{\Delta }(\epsilon ) \chi _{\Delta }(\epsilon )\right)$ at and a radius $\curlyvee$, in contrast to a point in the spherical fuzzy interpretation triangle as seen in typical SFSs. This novel set is an advancement beyond the standard SFS, as denoted by $\Delta = \Delta _o = \{\langle \epsilon , \xi _{\Delta }(\epsilon ), \eta _{\Delta }(\epsilon ), \chi _{\Delta }(\epsilon ); 0 \rangle \}$. However, the C-SFS with $\curlyvee (\epsilon ) > 0$ does not conform to a normal SFS.

The circular spherical fuzzy set features a consistent fixed radius across all elements. In contrast, the disc spherical fuzzy set exhibits distinct radii associated with each individual element.

#### Example 2.6

In the context where $\intercal =\{\epsilon _{1}, \epsilon _{2},\epsilon _{3}\}$, the C-SFS is exemplified as follows:

$$\begin{aligned} \Delta =\{(\epsilon _{1},0.4,0.4;0.6),(\epsilon _{2},0.7,0.1;0.6),(\epsilon _{3},0.2,0.3;0.6)\} \end{aligned}$$

#### Example 2.7

In the context where $\intercal =\{\epsilon _{1}, \epsilon _{2},\epsilon _{3}\}$, the D-SFS is exemplified as follows:

$$\begin{aligned} \Delta =\{(\epsilon _{1},0.2,0.4;0.6),(\epsilon _{2},0.3,0.9;0.2),(\epsilon _{3},0.4,0.5;0.9)\} \end{aligned}$$

### Formation of D-SFSs from SFSs^31^

This section shows how to calculate the radius of D-SFS and how to perform the SFS to D-SFS conversion.

To compute the $\curlyvee$ of D-SFS, apply equations listed below in this section. In an SFS $\digamma _{m}$, assuming SF pairings keep a structure $\{(\xi _{m,1}, \eta _{m,1}, \chi _{m,1}), (\xi _{m,2}, \eta _{m,2},\chi _{m,2}), (\xi _{m,3},\eta _{m,3}, \chi _{m,3})$ where *m* is a numerical value of SFS $\digamma _{m}$, typically comprising $\Pi _{m},$ which is a count of SF pairings $\digamma _{m}$. The arithematic average of the SF pairs can be determined with the following equation:

$$\begin{aligned} (\xi _{(\digamma _{m})}, \eta _{(\digamma _{m})},\chi _{(\digamma _{m})})= \left( \sqrt{\frac{\sum _{\Bbbk =1}^{\Pi _{m}}\xi _{m,\Bbbk }^{2}}{\Pi _{m}}},\sqrt{\frac{\sum _{\Bbbk =1}^{\Pi _{m}}\eta _{m,\Bbbk }^{2}}{\Pi _{m}}},\sqrt{\frac{\sum _{\Bbbk =1}^{\Pi _{m}}\chi _{m,\Bbbk }^{2}}{\Pi _{m}}}\right) \end{aligned}$$

The radius of $(\xi _{(\digamma _{m})}, \eta _{(\digamma _{m})},\chi _{(\digamma _{m})})$ is the largest value of Euclidean distance.

$$\begin{aligned} \curlyvee = \max \limits _{1 \le m \le \Pi _{m}}^{\phantom{0}} \sqrt{(\xi _{\digamma _{m}}-\xi _{m,n})^{2}+(\eta _{\digamma _{m}}-\eta _{m,n})^{2}+(\chi _{\digamma _{m}}-\chi _{m,n})^{2}} \end{aligned}$$

Thus, the SFS is undergoing conversion into D-SFS.

### Ranking of D-SFSs

In this section, the procedure for assigning rankings to our D-SFSs is outlined.

#### Definition 2.8

^31^ Consider $\Delta _{\flat }=\{(\xi _{\Delta _{\flat }} ,\eta _{\Delta _{\flat }},\chi _{\Delta _{\flat }} ;\curlyvee _{\Delta _{\flat }} |\epsilon \in \intercal )\}$ as any D-SFS.

1. Score Function: $\wp (\Delta )=\frac{1}{4}\left( \xi _{\Delta }-\eta _{\Delta }-\chi _{\Delta }+\sqrt{2\curlyvee }(2\aleph -1)\right)$ where $\wp {(\Delta )}\in [-1,1]$ and $\aleph \in [0,1]$
2. Accuracy Function: $\Im (\Delta )=\xi _{\Delta }^{2}+\eta _{\Delta }^{2}+\chi _{\Delta }^{2}$ and $\Im (\Delta ) \in [0,1].$

Now,assume $\Delta _{1}$ and $\Delta _{2}$ be two D-SFSs then,

- If $$\wp (\Delta _{1})>\wp (\Delta _{2})$$, then $$\Delta _{1} > \Delta _{2}$$
- If $$\wp (\Delta _{1})<\wp (\Delta _{2})$$, then $$\Delta _{1} < \Delta _{2}.$$

If $$\wp (\Delta _{1})=\wp (\Delta _{2})$$, then

- If $$\Im (\Delta _{1})>\Im (\Delta _{2})$$, then $$\Delta _{1} > \Delta _{2}$$
- If $$\Im (\Delta _{1})<\Im (\Delta _{2})$$, then $$\Delta _{1} < \Delta _{2}$$
- If $$\Im (\Delta _{1})=\Im (\Delta _{2})$$, then $$\Delta _{1} \approx \Delta _{2}.$$

### Aczel–Alsina norms

Within this part, we’ve showcased the Azcel-Alsina triangular norms.

#### Definition 2.9

^49^ A mapping $(\beth _{\mathfrak {B}}^{\mho })_{\mho \in [0,1]}$ is a Aczel–Alsina $\mathcal{T}\mathcal{N}$ if

$$\begin{aligned} \beth _{\mathfrak {B}}^{\mho }(\wp ,\partial )=\left\{ \begin{array}{ll} \beth _{\mathfrak {C}}(\wp ,\partial ), &{}\quad {\text {if}} ~ \mho = 0,\\ min(\wp ,\partial ), &{}\quad {\text {if}} ~\mho \rightarrow \infty ,\\ e^{-\left( (\ln ^{\backprime }\wp )^{\mho }+(\ln ^{\backprime }\partial )^{\mho }\right) ^{\frac{1}{\mho }}}, &{}\quad {\text {otherwise}} \\ \end{array} \right. \end{aligned}$$

In this context, where $\wp$ and $\partial$ are constrained within the interval [0,1], $\mho$ is a positive fixed value, and $\beth _{\mathfrak {C}}$ denotes an extreme t-norm.

#### Definition 2.10

^49^ A mapping $(\daleth _{\mathfrak {B}}^{\mho })_{\mho \in [0,1]}$ is a Aczel–Alsina $\mathcal {TCN}$ if

$$\begin{aligned} \daleth _{\mathfrak {B}}^{\mho }(\wp ,\partial )=\left\{ \begin{array}{ll} \daleth _{\mathfrak {C}}(\wp ,\partial ), &{}\quad {\text { if}} ~ \mho = 0,\\ max(\wp ,\partial ), &{}\quad {\text {if}} ~\mho ~ \rightarrow \infty ,\\ 1-e^{-\left( (\ln ^{\backprime }(1-\wp ))^{\mho }+(\ln ^{\backprime }(1-\partial ))^{\mho }\right) ^{\frac{1}{\mho }}}, &{}\quad {\text {otherwise}}\\ \end{array} \right. \end{aligned}$$

In this context, where $\wp$ and $\partial$ lie in the range [0,1], and $\mho$ is a positive fixed value, $\beth _{\mathfrak {C}}$ represents an extreme s-norm.

The utilization of Aczel Alsina aggregation operators in decision-making is not novel^50,51^. However, in this study, we are pioneering their application within the context of D-SFS methodology.

## Exploring Azcel–Alsina operations in D-SFSs

In this section, we delve into Azcel-Alsina operations, exploring their connection with fundamental properties. We also present theorems along with detailed proofs, enriching the understanding of their significance.

### Definition 3.1

Consider two D-SFSs $\Delta _{\flat }=\{\xi _{\Delta _{\flat }}, \eta _{\Delta _{\flat }}, \chi _{\Delta _{\flat }}; \curlyvee _{\Delta _{\flat }}\}$ where $\flat =\{1,2\}$. Then A-A norms operations are given as:

1. $$\begin{array}{c} \Delta _{1} \oplus _{\min } \Delta _{2}=\left( \begin{array}{c} \left( 1-e^{-\left( \big (-ln^{\backprime }(1-\xi _{\Delta _{1}}^{2})\big )^{\mho }+ \big (-ln^{\backprime }(1-\xi _{\Delta _{2}}^{2})\big )^{\mho }\right) ^\frac{1}{\mho }}\right) ^{\frac{1}{2}},\\ \left( e^{-\left( \big (-ln^{\backprime }\eta _{\Delta _{1}}^{2}\big )^{\mho }+ \big (-ln^{\backprime }\eta _{\Delta _{2}}^{2}\big )^{\mho }\right) ^\frac{1}{\mho }}\right) ^{\frac{1}{2}},\\ \left( e^{-\left( \big (-ln^{\backprime }\chi _{\Delta _{1}}^{2}\big )^{\mho }+ \big (-ln^{\backprime }\chi _{\Delta _{2}}^{2}\big )^{\mho }\right) ^\frac{1}{\mho }}\right) ^{\frac{1}{2}};\\ \left( e^{-\left( \big (-ln^{\backprime }\curlyvee _{\Delta _{1}}^{2}\big )^{\mho }+ \big (-ln^{\backprime }\curlyvee _{\Delta _{2}}^{2}\big )^{\mho }\right) ^\frac{1}{\mho }}\right) ^{\frac{1}{2}} \end{array} \right) \end{array}$$
2. $$\begin{array}{c} \Delta _{1} \oplus _{\max } \Delta _{2}=\left( \begin{array}{c} \left( 1-e^{-\left( \big (-ln^{\backprime }(1-\xi _{\Delta _{1}}^{2})\big )^{\mho }+ \big (-ln^{\backprime }(1-\xi _{\Delta _{2}}^{2})\big )^{\mho }\right) ^\frac{1}{\mho }}\right) ^{\frac{1}{2}},\\ \left( e^{-\left( \big (-ln^{\backprime }\eta _{\Delta _{1}}^{2}\big )^{\mho }+ \big (-ln^{\backprime }\eta _{\Delta _{2}}^{2}\big )^{\mho }\right) ^\frac{1}{\mho }}\right) ^{\frac{1}{2}},\\ \left( e^{-\left( \big (-ln^{\backprime }\chi _{\Delta _{1}}^{2}\big )^{\mho }+ \big (-ln^{\backprime }\chi _{\Delta _{2}}^{2}\big )^{\mho }\right) ^\frac{1}{\mho }}\right) ^{\frac{1}{2}};\\ \left( 1-e^{-\left( \big (-ln^{\backprime }(1-\curlyvee _{\Delta _{1}}^{2})\big )^{\mho }+ \big (-ln^{\backprime }(1-\curlyvee _{\Delta _{2}}^{2})\big )^{\mho }\right) ^\frac{1}{\mho }}\right) ^{\frac{1}{2}} \end{array} \right) \end{array}$$
3. $$\begin{array}{c} \Delta _{1} \otimes _{\min } \Delta _{2}=\left( \begin{array}{c} \left( e^{-\left( \big (-ln^{\backprime }\xi _{\Delta _{1}}^{2}\big )^{\mho }+ \big (-ln^{\backprime }\xi _{\Delta _{2}}^{2}\big )^{\mho }\right) ^\frac{1}{\mho }}\right) ^{\frac{1}{2}}\\ \left( e^{-\left( \big (-ln^{\backprime }\eta _{\Delta _{1}}^{2}\big )^{\mho }+ \big (-ln^{\backprime }\eta _{\Delta _{2}}^{2}\big )^{\mho }\right) ^\frac{1}{\mho }}\right) ^{\frac{1}{2}},\\ \left( 1-e^{-\left( \big (-ln^{\backprime }(1-\chi _{\Delta _{1}}^{2})\big )^{\mho }+ \big (-ln^{\backprime }(1-\chi _{\Delta _{2}}^{2})\big )^{\mho }\right) ^\frac{1}{\mho }}\right) ^{\frac{1}{2}}; \\ \left( e^{-\left( \big (-ln^{\backprime }\curlyvee _{\Delta _{1}}^{2}\big )^{\mho }+ \big (-ln^{\backprime }\curlyvee _{\Delta _{2}}^{2}\big )^{\mho }\right) ^\frac{1}{\mho }}\right) ^{\frac{1}{2}} \end{array} \right) \end{array}$$
4. $$\begin{array}{c} \Delta _{1} \otimes _{\max } \Delta _{2}=\left( \begin{array}{c} \left( e^{-\left( \big (-ln^{\backprime }\xi _{\Delta _{1}}^{2}\big )^{\mho }+ \big (-ln^{\backprime }\xi _{\Delta _{2}}^{2}\big )^{\mho }\right) ^\frac{1}{\mho }}\right) ^{\frac{1}{2}}\\ \left( e^{-\left( \big (-ln^{\backprime }\eta _{\Delta _{1}}^{2}\big )^{\mho }+ \big (-ln^{\backprime }\eta _{\Delta _{2}}^{2}\big )^{\mho }\right) ^\frac{1}{\mho }}\right) ^{\frac{1}{2}},\\ \left( 1-e^{-\left( \big (-ln^{\backprime }(1-\chi _{\Delta _{1}}^{2})\big )^{\mho }+ \big (-ln^{\backprime }(1-\chi _{\Delta _{2}}^{2})\big )^{\mho }\right) ^\frac{1}{\mho }}\right) ^{\frac{1}{2}}; \\ \left( 1-e^{-\left( \big (-ln^{\backprime }(1-\curlyvee _{\Delta _{1}}^{2})\big )^{\mho }+ \big (-ln^{\backprime }(1-\curlyvee _{\Delta _{2}}^{2})\big )^{\mho }\right) ^\frac{1}{\mho }}\right) ^{\frac{1}{2}} \end{array} \right) \end{array}$$
5. $$\begin{array}{c} \psi . \Delta _{1}=\left( \begin{array}{c} \left( 1-e^{-\left( \psi \big (-ln^{\backprime }(1-\xi _{\Delta _{1}}^{2})\big )^{\mho }\right) ^\frac{1}{\mho }}\right) ^{\frac{1}{2}}, \left( e^{-\left( \psi \big (-ln^{\backprime }\eta _{\Delta _{1}}^{2}\big )^{\mho }\right) ^\frac{1}{\mho }}\right) ^{\frac{1}{2}},\\ \left( e^{-\left( \psi \big (-ln^{\backprime }\chi _{\Delta _{1}}^{2}\big )^{\mho }\right) ^\frac{1}{\mho }}\right) ^{\frac{1}{2}}; \left( e^{-\left( \psi \big (-ln^{\backprime }\curlyvee _{\Delta _{1}}^{2}\big )^{\mho }\right) ^\frac{1}{\mho }}\right) ^{\frac{1}{2}} \end{array} \right) \end{array}$$
6. $$\begin{array}{c} \Delta _{1}^{\psi }=\left( \begin{array}{c} \left( e^{-\left( \psi \big (-ln^{\backprime }\xi _{\Delta _{1}}^{2}\big )^{\mho }\right) ^\frac{1}{\mho }}\right) ^{\frac{1}{2}}, \left( e^{-\left( \psi \big (-ln^{\backprime }\eta _{\Delta _{1}}^{2}\big )^{\mho }\right) ^\frac{1}{\mho }}\right) ^{\frac{1}{2}},\\ \left( 1-e^{-\left( \psi \big (-ln^{\backprime }(1-\chi _{\Delta _{1}}^{2})\big )^{\mho }\right) ^\frac{1}{\mho }}\right) ^{\frac{1}{2}}; \left( e^{-\left( \psi \big (-ln^{\backprime }\curlyvee _{\Delta _{1}}^{2}\big )^{\mho }\right) ^\frac{1}{\mho }}\right) ^{\frac{1}{2}} \end{array} \right) \end{array}$$

### Theorem 3.2

Assume three D-SFSs are $\Delta _{1}=(\xi _{\Delta _{1}} ,\eta _{\Delta _{1}},\chi _{\Delta _{1}} ;\curlyvee _{\Delta _{1}}),\Delta _{2}=(\xi _{\Delta _{2}} ,\eta _{\Delta _{2}},\chi _{\Delta _{2}} ;\curlyvee _{\Delta _{2}} )$ and $\Delta _{3}=(\xi _{\Delta _{3}} ,\eta _{\Delta _{3}},\chi _{\Delta _{3}} ;\curlyvee _{\Delta _{3}}).$ The ensuing properties are duly satisfied.

1. $$\Delta _{1}+\Delta _{2}=\Delta _{2}+ \Delta _{1}$$
2. $$\Delta _{1}\times \Delta _{2}=\Delta _{2}\times \Delta _{1}$$
3. $$(\Delta _{1}+\Delta _{2}) + \Delta _{3}=\Delta _{1}+(\Delta _{2} +\Delta _{3})$$
4. $$(\Delta _{1}\times \Delta _{2}) \times \Delta _{3}=\Delta _{1}\times (\Delta _{2} \times \Delta _{3})$$
5. $$*\Delta _{1}+*\Delta _{2} =*(\Delta _{1}+\Delta _{2}),~~ *> 0;$$
6. $$*_{\Delta _{1}}\Delta _{1}+*_{\Delta _{2}}\Delta _{1} =(*_{\Delta _{1}}+*_{\Delta _{2}})\Delta _{1},~~ *_{\Delta _{1}}$$ and $$*_{\Delta _{2}}> 0;$$
7. $$(\Delta _{1} \times \Delta _{2})^{*}=\Delta _{1}^{*} \times \Delta _{2}^{*},~~ *> 0$$
8. $$\Delta _{1}^{*_{\Delta _{1}}} \times \Delta _{1}^{*_{\Delta _{2}}}=\Delta _{1}^{*_{\Delta _{1}}+*_{\Delta _{2}}},~~ *_{\Delta _{1}}$$ and $$*_{\Delta _{2}}> 0$$

### Proof

**(1).**

To prove, $$\Delta _{1}\oplus _{min} \Delta _{2}=\Delta _{2} \oplus _{min} \Delta _{1}$$.

Consider,

$$\begin{aligned}{} & {} L.H.S=\Delta _{1}\oplus _{min} \Delta _{2}\\{} & {} \quad =(\xi _{\Delta _{1}},\eta _{\Delta _{1}},\chi _{\Delta _{1}};\curlyvee _{\Delta _{1}})\oplus (\xi _{\Delta _{2}},\eta _{\Delta _{2}},\chi _{\Delta _{2}};\curlyvee _{\Delta _{1}}))\\{} & {} \quad \begin{array}{c}=\left( \begin{array}{c} \left( 1-e^{-\left( \big (-ln^{\backprime }(1-\xi _{\Delta _{1}}^{2})\big )^{\mho }+ \big (-ln^{\backprime }(1-\xi _{\Delta _{2}}^{2})\big )^{\mho }\right) ^\frac{1}{\mho }}\right) ^{\frac{1}{2}}, \left( e^{-\left( \big (-ln^{\backprime }\eta _{\Delta _{1}}^{2}\big )^{\mho }+ \big (-ln^{\backprime }\eta _{\Delta _{2}}^{2}\big )^{\mho }\right) ^\frac{1}{\mho }}\right) ^{\frac{1}{2}},\\ \left( e^{-\left( \big (-ln^{\backprime }\chi _{\Delta _{1}}^{2}\big )^{\mho }+ \big (-ln^{\backprime }\chi _{\Delta _{2}}^{2}\big )^{\mho }\right) ^\frac{1}{\mho }}\right) ^{\frac{1}{2}}; \left( e^{-\left( \big (-ln^{\backprime }\curlyvee _{\Delta _{1}}^{2}\big )^{\mho }+ \big (-ln^{\backprime }\curlyvee _{\Delta _{2}}^{2}\big )^{\mho }\right) ^\frac{1}{\mho }}\right) ^{\frac{1}{2}} \end{array} \right) . \end{array}\\{} & {} \quad \begin{array}{c} =\left( \begin{array}{c} \left( 1-e^{-\left( \big (-ln^{\backprime }(1-\xi _{\Delta _{2}}^{2})\big )^{\mho }+ \big (-ln^{\backprime }(1-\xi _{\Delta _{1}}^{2})\big )^{\mho }\right) ^\frac{1}{\mho }}\right) ^{\frac{1}{2}}, \left( e^{-\left( \big (-ln^{\backprime }\eta _{\Delta _{2}}^{2}\big )^{\mho }+ \big (-ln^{\backprime }\eta _{\Delta _{1}}^{2}\big )^{\mho }\right) ^\frac{1}{\mho }}\right) ^{\frac{1}{2}},\\ \left( e^{-\left( \big (-ln^{\backprime }\chi _{\Delta _{2}}^{2}\big )^{\mho }+ \big (-ln^{\backprime }\chi _{\Delta _{1}}^{2}\big )^{\mho }\right) ^\frac{1}{\mho }}\right) ^{\frac{1}{2}}; \left( e^{-\left( \big (-ln^{\backprime }\curlyvee _{\Delta _{2}}^{2}\big )^{\mho }+ \big (-ln^{\backprime }\curlyvee _{\Delta _{1}}^{2}\big )^{\mho }\right) ^\frac{1}{\mho }}\right) ^{\frac{1}{2}} \end{array} \right) . \end{array}\\{} & {} \quad =\Delta _{2} \oplus _{min} \Delta _{1}=R.H.S \end{aligned}$$

Hence proved. $$\square$$

### Proof

**(2).** To prove, $$\Delta _{1}\otimes _{min} \Delta _{2}=\Delta _{2} \otimes _{min} \Delta _{1}$$.

Consider,

$$\begin{aligned}{} & {} L.H.S=\Delta _{1}\otimes _{min} \Delta _{2}\\{} & {} \quad =(\xi _{\Delta _{1}},\eta _{\Delta _{1}},\chi _{\Delta _{1}};\curlyvee _{\Delta _{1}})\otimes (\xi _{\Delta _{2}},\eta _{\Delta _{2}},\chi _{\Delta _{2}};\curlyvee _{\Delta _{2}})\\{} & {} \quad \begin{array}{c} =\left( \begin{array}{c} \left( e^{-\left( \big (-ln^{\backprime }\xi _{\Delta _{1}}^{2}\big )^{\mho }+ \big (-ln^{\backprime }\xi _{\Delta _{2}}^{2}\big )^{\mho }\right) ^\frac{1}{\mho }}\right) ^{\frac{1}{2}}, \left( e^{-\left( \big (-ln^{\backprime }\eta _{\Delta _{1}}^{2}\big )^{\mho }+ \big (-ln^{\backprime }\eta _{\Delta _{2}}^{2}\big )^{\mho }\right) ^\frac{1}{\mho }}\right) ^{\frac{1}{2}},\\ \left( 1-e^{-\left( \big (-ln^{\backprime }(1-\chi _{\Delta _{1}}^{2})\big )^{\mho }+ \big (-ln^{\backprime }(1-\chi _{\Delta _{2}}^{2})\big )^{\mho }\right) ^\frac{1}{\mho }}\right) ^{\frac{1}{2}}; \left( e^{-\left( \big (-ln^{\backprime }\curlyvee _{\Delta _{1}}^{2}\big )^{\mho }+ \big (-ln^{\backprime }\curlyvee _{\Delta _{2}}^{2}\big )^{\mho }\right) ^\frac{1}{\mho }}\right) ^{\frac{1}{2}} \end{array} \right) . \end{array}\\{} & {} \quad \begin{array}{c} =\left( \begin{array}{c} \left( e^{-\left( \big (-ln^{\backprime }\xi _{\Delta _{2}}^{2}\big )^{\mho }+ \big (-ln^{\backprime }\xi _{\Delta _{1}}^{2}\big )^{\mho }\right) ^\frac{1}{\mho }}\right) ^{\frac{1}{2}}, \left( e^{-\left( \big (-ln^{\backprime }\eta _{\Delta _{2}}^{2}\big )^{\mho }+ \big (-ln^{\backprime }\eta _{\Delta _{1}}^{2}\big )^{\mho }\right) ^\frac{1}{\mho }}\right) ^{\frac{1}{2}},\\ \left( 1-e^{-\left( \big (-ln^{\backprime }(1-\chi _{\Delta _{2}}^{2})\big )^{\mho }+ \big (-ln^{\backprime }(1-\chi _{\Delta _{1}}^{2})\big )^{\mho }\right) ^\frac{1}{\mho }}\right) ^{\frac{1}{2}}; \left( e^{-\left( \big (-ln^{\backprime }\curlyvee _{\Delta _{2}}^{2}\big )^{\mho }+ \big (-ln^{\backprime }\curlyvee _{\Delta _{1}}^{2}\big )^{\mho }\right) ^\frac{1}{\mho }}\right) ^{\frac{1}{2}} \end{array} \right) . \end{array}\\{} & {} \quad =\Delta _{2} \otimes _{min} \Delta _{1}=R.H.S \end{aligned}$$

Hence proved. $$\square$$

### Proof

**(5).** To prove, $*\psi _{1}\oplus *\Delta _{2} =*(\Delta _{1}\oplus \Delta _{2})$. Consider,

$$\begin{aligned}{} & {} L.H.S= *\Delta _{1}\oplus *\Delta _{2}\\{} & {} \quad \begin{array}{c} =\left( \begin{array}{c} \left( 1-e^{-\left( *\big (-ln^{\backprime }(1-\xi _{\Delta _{1}}^{2})\big )^{\mho }\right) ^\frac{1}{\mho }}\right) ^{\frac{1}{2}}, \left( e^{-\left( *\big (-ln^{\backprime }\eta _{\Delta _{1}}^{2}\big )^{\mho }\right) ^\frac{1}{\mho }}\right) ^{\frac{1}{2}},\\ \left( e^{-\left( *\big (-ln^{\backprime }\chi _{\Delta _{1}}^{2}\big )^{\mho }\right) ^\frac{1}{\mho }}\right) ^{\frac{1}{2}}; \left( e^{-\left( *\big (-ln^{\backprime }\curlyvee _{\Delta _{1}}^{2}\big )^{\mho }\right) ^\frac{1}{\mho }}\right) ^{\frac{1}{2}} \end{array} \right) \end{array}\\{} & {} \quad \begin{array}{c} \oplus \left( \begin{array}{c} \left( 1-e^{-\left( *\big (-ln^{\backprime }(1-\xi _{\Delta _{2}}^{2})\big )^{\mho }\right) ^\frac{1}{\mho }}\right) ^{\frac{1}{2}}, \left( e^{-\left( *\big (-ln^{\backprime }\eta _{\Delta _{2}}^{2}\big )^{\mho }\right) ^\frac{1}{\mho }}\right) ^{\frac{1}{2}},\\ \left( e^{-\left( *\big (-ln^{\backprime }\chi _{\Delta _{2}}^{2}\big )^{\mho }\right) ^\frac{1}{\mho }}\right) ^{\frac{1}{2}}; \left( e^{-\left( *\big (-ln^{\backprime }\curlyvee _{\Delta _{2}}^{2}\big )^{\mho }\right) ^\frac{1}{\mho }}\right) ^{\frac{1}{2}} \end{array} \right) \end{array}\\{} & {} \quad \begin{array}{c} =\left( \begin{array}{c} \left( 1-e^{-\left( \left( \left( *\big (-ln^{\backprime }(1-\xi _{\Delta _{1}}^{2})\big )^{\mho }\right) ^\frac{1}{\mho }\right) ^{\mho }+\left( \left( *\big (-ln^{\backprime }(1-\xi _{\Delta _{2}}^{2})\big )^{\mho }\right) ^\frac{1}{\mho }\right) ^{\mho } \right) ^\frac{1}{\mho }}\right) ^{\frac{1}{2}},\\ \left( e^{-\left( \left( \left( *\big (-ln^{\backprime }\eta _{\Delta _{1}}^{2}\big )^{\mho }\right) ^\frac{1}{\mho }\right) ^{\mho }+ \left( \left( *\big (-ln^{\backprime }\eta _{\Delta _{1}}^{2}\big )^{\mho }\right) ^\frac{1}{\mho }\right) ^{\mho }\right) ^\frac{1}{\mho }}\right) ^{\frac{1}{2}},\\ \left( e^{-\left( \left( \left( *\big (-ln^{\backprime }\chi _{\Delta _{1}}^{2}\big )^{\mho }\right) ^\frac{1}{\mho }\right) ^{\mho }+ \left( \left( *\big (-ln^{\backprime }\chi _{\Delta _{1}}^{2}\big )^{\mho }\right) ^\frac{1}{\mho }\right) ^{\mho }\right) ^\frac{1}{\mho }}\right) ^{\frac{1}{2}},\\ \left( e^{-\left( \left( \left( *\big (-ln^{\backprime }\curlyvee _{\Delta _{1}}^{2}\big )^{\mho }\right) ^\frac{1}{\mho }\right) ^{\mho }+ \left( \left( *\big (-ln^{\backprime }\curlyvee _{\Delta _{1}}^{2}\big )^{\mho }\right) ^\frac{1}{\mho }\right) ^{\mho }\right) ^\frac{1}{\mho }}\right) ^{\frac{1}{2}} \end{array} \right) \end{array}\\{} & {} \quad \begin{array}{c} =\left( \begin{array}{c} \left( 1-e^{-\left( *\big (-ln^{\backprime }(1-\xi _{\Delta _{1}}^{2})\big )^{\mho }+ *\big (-ln^{\backprime }(1-\xi _{\Delta _{2}}^{2})\big )^{\mho }\right) ^\frac{1}{\mho }}\right) ^{\frac{1}{2}}, \left( e^{-\left( *\big (-ln^{\backprime }\eta _{\Delta _{1}}^{2}\big )^{\mho }+ *\big (-ln^{\backprime }\eta _{\Delta _{2}}^{2}\big )^{\mho }\right) ^\frac{1}{\mho }}\right) ^{\frac{1}{2}},\\ \left( e^{-\left( *\big (-ln^{\backprime }\chi _{\Delta _{1}}^{2}\big )^{\mho }+ *\big (-ln^{\backprime }\chi _{\Delta _{2}}^{2}\big )^{\mho }\right) ^\frac{1}{\mho }}\right) ^{\frac{1}{2}}; \left( e^{-\left( *\big (-ln^{\backprime }\curlyvee _{\Delta _{1}}^{2}\big )^{\mho }+ *\big (-ln^{\backprime }\curlyvee _{\Delta _{2}}^{2}\big )^{\mho }\right) ^\frac{1}{\mho }}\right) ^{\frac{1}{2}} \end{array} \right) \end{array}\\{} & {} \quad \begin{array}{c} =\left( \begin{array}{c} \left( 1-e^{-\left( *\left( \big (-ln^{\backprime }(1-\xi _{\Delta _{1}}^{2})\big )^{\mho }+ \big (-ln^{\backprime }(1-\xi _{\Delta _{2}}^{2})\big )^{\mho }\right) \right) ^\frac{1}{\mho }}\right) ^{\frac{1}{2}}, \left( e^{-\left( *\left( \big (-ln^{\backprime }\eta _{\Delta _{1}}^{2}\big )^{\mho }+ \big (-ln^{\backprime }\eta _{\Delta _{2}}^{2}\big )^{\mho }\right) \right) ^\frac{1}{\mho }}\right) ^{\frac{1}{2}},\\ \left( e^{-\left( *\left( \big (-ln^{\backprime }\chi _{\Delta _{1}}^{2}\big )^{\mho }+ \big (-ln^{\backprime }\chi _{\Delta _{2}}^{2}\big )^{\mho }\right) \right) ^\frac{1}{\mho }}\right) ^{\frac{1}{2}}; \left( e^{-\left( *\left( \big (-ln^{\backprime }\curlyvee _{\Delta _{1}}^{2}\big )^{\mho }+ \big (-ln^{\backprime }\curlyvee _{\Delta _{2}}^{2}\big )^{\mho }\right) \right) ^\frac{1}{\mho }}\right) ^{\frac{1}{2}} \end{array} \right) \end{array}\\{} & {} \quad \begin{array}{c} =*\left( \begin{array}{c} \left( 1-e^{-\left( \big (-ln^{\backprime }(1-\xi _{\Delta _{1}}^{2})\big )^{\mho }+ \big (-ln^{\backprime }(1-\xi _{\Delta _{2}}^{2})\big )^{\mho }\right) ^\frac{1}{\mho }}\right) ^{\frac{1}{2}}, \left( e^{-\left( \big (-ln^{\backprime }\eta _{\Delta _{1}}^{2}\big )^{\mho }+ \big (-ln^{\backprime }\eta _{\Delta _{2}}^{2}\big )^{\mho }\right) ^\frac{1}{\mho }}\right) ^{\frac{1}{2}},\\ \left( e^{-\left( \big (-ln^{\backprime }\chi _{\Delta _{1}}^{2}\big )^{\mho }+ \big (-ln^{\backprime }\chi _{\Delta _{2}}^{2}\big )^{\mho }\right) ^\frac{1}{\mho }}\right) ^{\frac{1}{2}}; \left( e^{-\left( \big (-ln^{\backprime }\curlyvee _{\Delta _{1}}^{2}\big )^{\mho }+ \big (-ln^{\backprime }\curlyvee _{\Delta _{2}}^{2}\big )^{\mho }\right) ^\frac{1}{\mho }}\right) ^{\frac{1}{2}} \end{array} \right) \end{array}\\{} & {} \quad =*(\Delta _{1}\oplus \Delta _{2}) \end{aligned}$$

As R.H.S=L.H.S, hence proved. $\square$

## Aggregation operators for D-SFS

This section introduces the topic of aggregation operations for disc spherical fuzzy sets (D-SFSs). We will explore the mathematical foundations of these operators and elucidate several fundamental characteristics they possess. Furthermore, we will provide comprehensive justifications for the accuracy and reliability of these operators in amalgamating D-SFS data. The introduction of this method aims to enhance the understanding of how Azcel-Alsina norms can be effectively employed in aggregation operations for D-SFSs.

### Exploring D-SFS Aczel–Alsina averaging aggregation operators

In this specific subsection, we have presented and explained the concept of a weighted averaging aggregation operation applied to D-SFSs. The aggregation process involves utilizing the Azcel-Alsina norm, a specific mathematical framework, to combine information from different D-SFSs. The weighted averaging operation considers the influence of each element proportionally based on assigned weights, providing a more nuanced and flexible approach to data integration in the context of D-SFSs.

#### Definition 4.1

Consider the collection of D-SFNs denoted as $\Delta _{\flat }=\{\xi _{\Delta _{\flat }}, \eta _{\Delta _{\flat }}, \chi _{\Delta _{\flat }}; \curlyvee _{\Delta _{\flat }}\}$, where $\flat =\{1,2,\ldots ,\ell \}$. A D-SN Azcel-Alsina weighted averaging (D-SFAAWA) AO of dimension $\ell$ is a mapping characterized by a weight vector $\Theta =(\Theta _{1},\Theta _{2},\ldots ,\Theta _{\ell })$, ensuring that $\Theta _{\flat } >0$ and $\sum _{\flat =1}^{\ell } \Theta _{\flat }=1$. The operation is defined as follows:

$$\begin{aligned} D-SFAAWA(\Delta _{1},\Delta _{2},\ldots ,\Delta _{\ell }) = \sum _{\flat =1}^{\ell } \Theta _{\flat } \Delta _{\flat } \end{aligned}$$

#### Theorem 4.2

Assume the collection of D-SFNs as $\Delta _{\flat }=\{\xi _{\Delta _{\flat }}, \eta _{\Delta _{\flat }}, \chi _{\Delta _{\flat }}; \curlyvee _{\Delta _{\flat }}\}$, where $\flat =\{1,2,\ldots ,\ell \}$. A D-SFN Azcel-Alsina weighted averaging (D-SFAAWA) Aggregation Operator of dimension $\ell$ is a mapping characterized by a weight vector $\Theta =(\Theta _{1},\Theta _{2},\ldots ,\Theta _{\ell })$, ensuring that $\Theta _{\flat } >0$ and $\sum _{\flat =1}^{\ell } \Theta _{\flat }=1$ is defined as follows:

$$\begin{aligned}{} & {} D-SFAAWA(\Delta _{1},\Delta _{2},\ldots ,\Delta _{\ell })=\\{} & {} \quad \begin{array}{c} \left( \begin{array}{c} \left( 1-e^{-\left( \sum _{\flat =1}^{\ell }\Theta _{\flat }\big (-ln^{\backprime }(1-\xi _{\Delta _{\flat }}^{2})\big )^{\mho }\right) ^\frac{1}{\mho }}\right) ^{\frac{1}{2}}, \left( e^{-\left( \sum _{\flat =1}^{\ell }\Theta _{\flat }\big (-ln^{\backprime }\eta _{\Delta _{\flat }}^{2}\big )^{\mho }\right) ^\frac{1}{\mho }}\right) ^{\frac{1}{2}},\\ \left( e^{-\left( \sum _{\flat =1}^{\ell }\Theta _{\flat }\big (-ln^{\backprime }\chi _{\Delta _{\flat }}^{2}\big )^{\mho }\right) ^\frac{1}{\mho }}\right) ^{\frac{1}{2}}; \left( e^{-\left( \sum _{\flat =1}^{\ell }\Theta _{\flat }\big (-ln^{\backprime }\curlyvee _{\Delta _{\flat }}^{2}\big )^{\mho }\right) ^\frac{1}{\mho }}\right) ^{\frac{1}{2}} \end{array} \right) \end{array} \end{aligned}$$

It is deducted that the obtained result is again a D-SFN.

#### Proof

The theorem is proven through the application of the induction method, which can be expressed as follows:

First, assume $\ell =2$, we have:

$$\begin{aligned}{} & {} D-SFAAWA(\Delta _{1},\Delta _{2},\ldots ,\Delta _{\ell })= \sum _{\flat =1}^{2}\Theta _{\flat }\Delta _{\flat }\\{} & {} \quad \begin{array}{c} \Theta _{1}\Delta _{1}=\left( \begin{array}{c} \left( 1-e^{-\left( \Theta _{1}\big (-ln^{\backprime }(1-\xi _{\Delta _{1}}^{2})\big )^{\mho }\right) ^\frac{1}{\mho }}\right) ^{\frac{1}{2}}, \left( e^{-\left( \Theta _{1}\big (-ln^{\backprime }\eta _{\Delta _{1}}^{2}\big )^{\mho }\right) ^\frac{1}{\mho }}\right) ^{\frac{1}{2}},\\ \left( e^{-\left( \Theta _{1}\big (-ln^{\backprime }\chi _{\Delta _{1}}^{2}\big )^{\mho }\right) ^\frac{1}{\mho }}\right) ^{\frac{1}{2}}; \left( e^{-\left( \Theta _{1}\big (-ln^{\backprime }\curlyvee _{\Delta _{1}}^{2}\big )^{\mho }\right) ^\frac{1}{\mho }}\right) ^{\frac{1}{2}} \end{array} \right) . \end{array}\\{} & {} \quad \begin{array}{c} \Theta _{2}\Delta _{2}=\left( \begin{array}{c} \left( 1-e^{-\left( \Theta _{2}\big (-ln^{\backprime }(1-\xi _{\Delta _{2}}^{2})\big )^{\mho }\right) ^\frac{1}{\mho }}\right) ^{\frac{1}{2}}, \left( e^{-\left( \Theta _{2}\big (-ln^{\backprime }\eta _{\Delta _{2}}^{2}\big )^{\mho }\right) ^\frac{1}{\mho }}\right) ^{\frac{1}{2}},\\ \left( e^{-\left( \Theta _{2}\big (-ln^{\backprime }\chi _{\Delta _{2}}^{2}\big )^{\mho }\right) ^\frac{1}{\mho }}\right) ^{\frac{1}{2}}; \left( e^{-\left( \Theta _{2}\big (-ln^{\backprime }\curlyvee _{\Delta _{2}}^{2}\big )^{\mho }\right) ^\frac{1}{\mho }}\right) ^{\frac{1}{2}} \end{array} \right) . \end{array} \end{aligned}$$

Now, applying the defined Aczel–Alsina operation, we have

$$\begin{aligned}{} & {} D-SFAAWA(\Delta _{1},\Delta _{2},\ldots ,\Delta _{\ell })= \Theta _{1}\Delta _{1}\oplus \Theta _{2}\Delta _{2}=\\{} & {} \quad \begin{array}{c} \left( \begin{array}{c} \left( 1-e^{-\left( \Theta _{1}\big (-ln^{\backprime }(1-\xi _{\Delta _{1}}^{2})\big )^{\mho }\right) ^\frac{1}{\mho }}\right) ^{\frac{1}{2}}, \left( e^{-\left( \Theta _{1}\big (-ln^{\backprime }\eta _{\Delta _{1}}^{2}\big )^{\mho }\right) ^\frac{1}{\mho }}\right) ^{\frac{1}{2}},\\ \left( e^{-\left( \Theta _{1}\big (-ln^{\backprime }\chi _{\Delta _{1}}^{2}\big )^{\mho }\right) ^\frac{1}{\mho }}\right) ^{\frac{1}{2}}; \left( e^{-\left( \Theta _{1}\big (-ln^{\backprime }\curlyvee _{\Delta _{1}}^{2}\big )^{\mho }\right) ^\frac{1}{\mho }}\right) ^{\frac{1}{2}} \end{array} \right) \end{array}\\{} & {} \quad \oplus \left( \begin{array}{c} \left( 1-e^{-\left( \Theta _{2}\big (-ln^{\backprime }(1-\xi _{\Delta _{2}}^{2})\big )^{\mho }\right) ^\frac{1}{\mho }}\right) ^{\frac{1}{2}}, \left( e^{-\left( \Theta _{2}\big (-ln^{\backprime }\eta _{\Delta _{2}}^{2}\big )^{\mho }\right) ^\frac{1}{\mho }}\right) ^{\frac{1}{2}},\\ \left( e^{-\left( \Theta _{2}\big (-ln^{\backprime }\chi _{\Delta _{2}}^{2}\big )^{\mho }\right) ^\frac{1}{\mho }}\right) ^{\frac{1}{2}}; 
\left( e^{-\left( \Theta _{2}\big (-ln^{\backprime }\curlyvee _{\Delta _{2}}^{2}\big )^{\mho }\right) ^\frac{1}{\mho }}\right) ^{\frac{1}{2}} \end{array} \right) \\{} & {} \quad \begin{array}{c} =\left( \begin{array}{c} \left( 1-e^{-\left( \Theta _{1}\big (-ln^{\backprime }(1-\xi _{\Delta _{1}}^{2})\big )^{\mho }+\Theta _{2}\big (-ln^{\backprime }(1-\xi _{\Delta _{2}}^{2})\big )^{\mho }\right) ^\frac{1}{\mho }}\right) ^{\frac{1}{2}}, \left( e^{-\left( \Theta _{1}\big (-ln^{\backprime }\eta _{\Delta _{1}}^{2}\big )^{\mho }+\Theta _{2}\big (-ln^{\backprime }\eta _{\Delta _{2}}^{2}\big )^{\mho }\right) ^\frac{1}{\mho }}\right) ^{\frac{1}{2}},\\ \left( e^{-\left( \Theta _{1}\big (-ln^{\backprime }\chi _{\Delta _{1}}^{2}\big )^{\mho }+\Theta _{2}\big (-ln^{\backprime }\chi _{\Delta _{2}}^{2}\big )^{\mho }\right) ^\frac{1}{\mho }}\right) ^{\frac{1}{2}}; \left( e^{-\left( \Theta _{1}\big (-ln^{\backprime }\curlyvee _{\Delta _{1}}^{2}\big )^{\mho }+\Theta _{2}\big (-ln^{\backprime }\curlyvee _{\Delta _{2}}^{2}\big )^{\mho }\right) ^\frac{1}{\mho }}\right) ^{\frac{1}{2}} \end{array} \right) \end{array}\\{} & {} \quad \begin{array}{c} =\left( \begin{array}{c} \left( 1-e^{-\left( \sum _{\flat =1}^{2}\Theta _{\flat }\big (-ln^{\backprime }(1-\xi _{\Delta _{\flat }}^{2})\big )^{\mho }\right) ^\frac{1}{\mho }}\right) ^{\frac{1}{2}}, \left( e^{-\left( \sum _{\flat =1}^{2}\Theta _{\flat }\big (-ln^{\backprime }\eta _{\Delta _{\flat }}^{2}\big )^{\mho }\right) ^\frac{1}{\mho }}\right) ^{\frac{1}{2}},\\ \left( e^{-\left( \sum _{\flat =1}^{2}\Theta _{\flat }\big (-ln^{\backprime }\chi _{\Delta _{\flat }}^{2}\big )^{\mho }\right) ^\frac{1}{\mho }}\right) ^{\frac{1}{2}}; \left( e^{-\left( \sum _{\flat =1}^{2}\Theta _{\flat }\big (-ln^{\backprime }\curlyvee _{\Delta _{\flat }}^{2}\big )^{\mho }\right) ^\frac{1}{\mho }}\right) ^{\frac{1}{2}} \end{array} \right) \end{array} \end{aligned}$$

Thus,the theorem holds true for $\ell =2$.

Now, assume that it is valid for $\ell =w$.

$$\begin{aligned}{} & {} D-SFAAWA(\Delta _{1},\Delta _{2},\ldots ,\Delta _{w})=\\{} & {} \quad \begin{array}{c} \left( \begin{array}{c} \left( 1-e^{-\left( \sum _{\flat =1}^{w}\Theta _{\flat }\big (-ln^{\backprime }(1-\xi _{\Delta _{\flat }}^{2})\big )^{\mho }\right) ^\frac{1}{\mho }}\right) ^{\frac{1}{2}}, \left( e^{-\left( \sum _{\flat =1}^{w}\Theta _{\flat }\big (-ln^{\backprime }\eta _{\Delta _{\flat }}^{2}\big )^{\mho }\right) ^\frac{1}{\mho }}\right) ^{\frac{1}{2}},\\ \left( e^{-\left( \sum _{\flat =1}^{w}\Theta _{\flat }\big (-ln^{\backprime }\chi _{\Delta _{\flat }}^{2}\big )^{\mho }\right) ^\frac{1}{\mho }}\right) ^{\frac{1}{2}}; \left( e^{-\left( \sum _{\flat =1}^{w}\Theta _{\flat }\big (-ln^{\backprime }\curlyvee _{\Delta _{\flat }}^{2}\big )^{\mho }\right) ^\frac{1}{\mho }}\right) ^{\frac{1}{2}} \end{array} \right) \end{array} \end{aligned}$$

Now, we’ll prove that it is valid for $\ell =w+1$.

That is,

$$\begin{aligned}{} & {} D-SFAAWA(\Delta _{1},\Delta _{2},\ldots ,\Delta _{w}, \Delta _{w+1})= \bigg (\sum _{\flat =1}^{w}\Theta _{w}\Delta _{w}\bigg )\oplus \big ( \Theta _{w+1}\Delta _{w+1}\big )=\\{} & {} \quad \begin{array}{c} \left( \begin{array}{c} \left( 1-e^{-\left( \sum _{\flat =1}^{w}\Theta _{\flat }\big (-ln^{\backprime }(1-\xi _{\Delta _{\flat }}^{2})\big )^{\mho }\right) ^\frac{1}{\mho }}\right) ^{\frac{1}{2}}, \left( e^{-\left( \sum _{\flat =1}^{w}\Theta _{\flat }\big (-ln^{\backprime }\eta _{\Delta _{\flat }}^{2}\big )^{\mho }\right) ^\frac{1}{\mho }}\right) ^{\frac{1}{2}},\\ \left( e^{-\left( \sum _{\flat =1}^{w}\Theta _{\flat }\big (-ln^{\backprime }\chi _{\Delta _{\flat }}^{2}\big )^{\mho }\right) ^\frac{1}{\mho }}\right) ^{\frac{1}{2}}; \left( e^{-\left( \sum _{\flat =1}^{w}\Theta _{\flat }\big (-ln^{\backprime }\curlyvee _{\Delta _{\flat }}^{2}\big )^{\mho }\right) ^\frac{1}{\mho }}\right) ^{\frac{1}{2}} \end{array} \right) \end{array}\\{} & {} \quad \oplus \left( \begin{array}{c} \left( 1-e^{-\left( \Theta _{w+1}\big (-ln^{\backprime }(1-\xi _{\Delta _{w+1}}^{2})\big )^{\mho }\right) ^\frac{1}{\mho }}\right) ^{\frac{1}{2}}, \left( e^{-\left( \Theta _{w+1}\big (-ln^{\backprime }\eta _{\Delta _{w+1}}^{2}\big )^{\mho }\right) ^\frac{1}{\mho }}\right) ^{\frac{1}{2}},\\ \left( e^{-\left( \Theta _{w+1}\big (-ln^{\backprime }\chi _{\Delta _{w+1}}^{2}\big )^{\mho }\right) ^\frac{1}{\mho }}\right) ^{\frac{1}{2}}; \left( e^{-\left( \Theta _{w+1}\big (-ln^{\backprime }\curlyvee _{\Delta _{w+1}}^{2}\big )^{\mho }\right) ^\frac{1}{\mho }}\right) ^{\frac{1}{2}} \end{array} \right) \\{} & {} \quad \begin{array}{c} =\left( \begin{array}{c} \left( 1-e^{-\left( \sum _{\flat =1}^{w}\Theta _{\flat }\big (-ln^{\backprime }(1-\xi _{\Delta _{\flat }}^{2})\big )^{\mho }+\Theta _{w+1}\big (-ln^{\backprime }(1-\xi _{\Delta _{w+1}}^{2})\big )^{\mho }\right) ^\frac{1}{\mho }}\right) ^{\frac{1}{2}},\\ \left( e^{-\left( \sum _{\flat =1}^{w}\Theta _{\flat }\big (-ln^{\backprime }\eta _{\Delta _{\flat }}^{2}\big )^{\mho }+\Theta _{w+1}\big (-ln^{\backprime }\eta _{\Delta _{w+1}}^{2}\big )^{\mho }\right) ^\frac{1}{\mho }}\right) ^{\frac{1}{2}},\\ \left( e^{-\left( \sum _{\flat =1}^{w}\Theta _{\flat }\big (-ln^{\backprime }\chi _{\Delta _{\flat }}^{2}\big )^{\mho }+\Theta _{w+1}\big (-ln^{\backprime }\chi _{\Delta _{w+1}}^{2}\big )^{\mho }\right) ^\frac{1}{\mho }}\right) ^{\frac{1}{2}};\\ \left( e^{-\left( \sum _{\flat =1}^{w}\Theta _{\flat }\big (-ln^{\backprime }\curlyvee _{\Delta _{\flat }}^{2}\big )^{\mho }+\Theta _{w+1}\big (-ln^{\backprime }\curlyvee _{\Delta _{w+1}}^{2}\big )^{\mho }\right) ^\frac{1}{\mho }}\right) ^{\frac{1}{2}} \end{array} \right) \end{array}\\{} & {} D-SFAAWA(\Delta _{1},\Delta _{2},\ldots ,\Delta _{w+1})=\\{} & {} \quad \begin{array}{c} \left( \begin{array}{c} \left( 1-e^{-\left( \sum _{\flat =1}^{w+1}\Theta _{\flat }\big (-ln^{\backprime }(1-\xi _{\Delta _{\flat }}^{2})\big )^{\mho }\right) ^\frac{1}{\mho }}\right) ^{\frac{1}{2}}, \left( e^{-\left( \sum _{\flat =1}^{w+1}\Theta _{\flat }\big (-ln^{\backprime }\eta _{\Delta _{\flat }}^{2}\big )^{\mho }\right) ^\frac{1}{\mho }}\right) ^{\frac{1}{2}},\\ \left( e^{-\left( \sum _{\flat =1}^{w+1}\Theta _{\flat }\big (-ln^{\backprime }\chi _{\Delta _{\flat }}^{2}\big )^{\mho }\right) ^\frac{1}{\mho }}\right) ^{\frac{1}{2}}; \left( e^{-\left( \sum _{\flat =1}^{w+1}\Theta _{\flat }\big (-ln^{\backprime }\curlyvee _{\Delta _{\flat }}^{2}\big )^{\mho }\right) ^\frac{1}{\mho }}\right) ^{\frac{1}{2}} \end{array} \right) \end{array} \end{aligned}$$

Hence, the statement of the theorem has been demonstrated to hold true for every possible value of $\ell$, confirming its validity across the entire range of applicable conditions. The proof process undertaken establishes the general applicability and correctness of the theorem, providing a comprehensive and conclusive verification. $\square$

Leveraging the D-SFAAWA operator enables us to efficiently highlight the distinctive characteristics outlined below.

#### Theorem 4.3

***Idempotency:***

Assuming $\Delta _{\flat }=\{\xi _{\Delta _{\flat }}, \eta _{\Delta _{\flat }}, \chi _{\Delta _{\flat }}; \curlyvee _{\Delta _{\flat }}\}$, where $\flat =\{1,2,\ldots ,\ell \}$ represents a set of equivalent D-SFNs, and $\Delta _{\flat }=\Delta$ holds for each $\flat =\{1,2,\ldots ,\ell \}$, then

6 $$\begin{aligned} D-SFAAWA\big (\Delta _{1},\Delta _{2},\ldots ,\Delta _{\ell }\big )=\Delta \end{aligned}$$

#### Proof

Since,

$$\begin{aligned}{} & {} D-SFAAWA(\Delta _{1},\Delta _{2},\ldots ,\Delta _{\ell })=\\{} & {} \quad \begin{array}{c} \left( \begin{array}{c} \left( 1-e^{-\left( \sum _{\flat =1}^{\ell }\Theta _{\flat }\big (-ln^{\backprime }(1-\xi _{\Delta _{\flat }}^{2})\big )^{\mho }\right) ^\frac{1}{\mho }}\right) ^{\frac{1}{2}}, \left( e^{-\left( \sum _{\flat =1}^{\ell }\Theta _{\flat }\big (-ln^{\backprime }\eta _{\Delta _{\flat }}^{2}\big )^{\mho }\right) ^\frac{1}{\mho }}\right) ^{\frac{1}{2}},\\ \left( e^{-\left( \sum _{\flat =1}^{\ell }\Theta _{\flat }\big (-ln^{\backprime }\chi _{\Delta _{\flat }}^{2}\big )^{\mho }\right) ^\frac{1}{\mho }}\right) ^{\frac{1}{2}}; \left( e^{-\left( \sum _{\flat =1}^{\ell }\Theta _{\flat }\big (-ln^{\backprime }\curlyvee _{\Delta _{\flat }}^{2}\big )^{\mho }\right) ^\frac{1}{\mho }}\right) ^{\frac{1}{2}} \end{array} \right) \end{array} \end{aligned}$$

Now, put $\Delta _{\flat }={\xi _{\Delta _{\flat }}, \eta _{\Delta _{\flat }}, \chi _{\Delta _{\flat }}; \curlyvee _{\Delta _{\flat }}}=\Delta$ where $\flat =\{1,2,\ldots ,\ell \}$.

$$\begin{aligned}{} & {} \begin{array}{c} D-SFAAWA(\Delta _{1},\Delta _{2},\ldots ,\Delta _{\ell })\left( \begin{array}{c} \left( 1-e^{-\left( \sum _{\flat =1}^{\ell }\Theta _{\flat }\big (-ln^{\backprime }(1-\xi _{\Delta }^{2})\big )^{\mho }\right) ^\frac{1}{\mho }}\right) ^{\frac{1}{2}}, \left( e^{-\left( \sum _{\flat =1}^{\ell }\Theta _{\flat }\big (-ln^{\backprime }\eta _{\Delta }^{2}\big )^{\mho }\right) ^\frac{1}{\mho }}\right) ^{\frac{1}{2}},\\ \left( e^{-\left( \sum _{\flat =1}^{\ell }\Theta _{\flat }\big (-ln^{\backprime }\chi _{\Delta }^{2}\big )^{\mho }\right) ^\frac{1}{\mho }}\right) ^{\frac{1}{2}}; \left( e^{-\left( \sum _{\flat =1}^{\ell }\Theta _{\flat }\big (-ln^{\backprime }\curlyvee _{\Delta }^{2}\big )^{\mho }\right) ^\frac{1}{\mho }}\right) ^{\frac{1}{2}} \end{array} \right) \end{array}\\{} & {} \quad \begin{array}{c} =\left( \begin{array}{c} \left( 1-e^{-\left( \big (-ln^{\backprime }(1-\xi _{\Delta }^{2})\big )^{\mho }\right) ^\frac{1}{\mho }}\right) ^{\frac{1}{2}}, \left( e^{-\left( \big (-ln^{\backprime }\eta _{\Delta }^{2}\big )^{\mho }\right) ^\frac{1}{\mho }}\right) ^{\frac{1}{2}},\\ \left( e^{-\left( \big (-ln^{\backprime }\chi _{\Delta }^{2}\big )^{\mho }\right) ^\frac{1}{\mho }}\right) ^{\frac{1}{2}}; \left( e^{-\left( \big (-ln^{\backprime }\curlyvee _{\Delta }^{2}\big )^{\mho }\right) ^\frac{1}{\mho }}\right) ^{\frac{1}{2}} \end{array} \right) \end{array}\\{} & {} \quad \{\xi _{\Delta }, \eta _{\Delta }, \chi _{\Delta }; \curlyvee _{\Delta }\}=\Delta . \end{aligned}$$

Thus, $$D-SFAAWA\big (\Delta _{1},\Delta _{2},\ldots ,\Delta _{\ell }\big )=\Delta$$ holds. $$\square$$

#### Theorem 4.4

***Boundedness:***

Assuming $\Delta _{\flat }=\{\xi _{\Delta _{\flat }}, \eta _{\Delta _{\flat }}, \chi _{\Delta _{\flat }}; \curlyvee _{\Delta _{\flat }}\}$, where $\flat =\{1,2,\ldots ,\ell \}$ represents a set of equivalent D-SFNs. Consider

$$\begin{aligned} \Delta _{\flat }^{+}=\bigg (\max _{\flat }\big \{\xi _{\Delta _{\flat }}\big \}, \min _{\flat }\big \{\eta _{\Delta _{\flat }}\big \}, \min _{\flat }\big \{\chi _{\Delta _{\flat }}\big \}; \min _{\flat }\big \{\curlyvee _{\Delta _{\flat }}\big \}\bigg ) \end{aligned}$$

and

$$\begin{aligned} \Delta _{\flat }^{-}=\bigg (\min _{\flat }\big \{\xi _{\Delta _{\flat }}\big \}, \min _{\flat }\big \{\eta _{\Delta _{\flat }}\big \}, \max _{\flat }\big \{\chi _{\Delta _{\flat }}\big \}; \min _{\flat }\big \{\curlyvee _{\Delta _{\flat }}\big \}\bigg ). \end{aligned}$$

Then,

7 $$\begin{aligned} \Delta _{\flat }^{-} \le D-SFAAWA\big (\Delta _{1},\Delta _{2},\ldots ,\Delta _{\ell }\big ) \le \Delta _{\flat }^{+}. \end{aligned}$$

#### Proof

Considering $\min _{\flat }\big \{\xi _{\Delta _{\flat }}\big \} \le \xi _{\Delta _{\flat }} \le \max _{\flat }\big \{\xi _{\Delta _{\flat }}\big \}$ it can be deduced that:

$$\begin{aligned} \begin{aligned} \left( 1-e^{-\left( \sum _{\flat =1}^{\ell }\Theta _{\flat }\big (-ln^{\backprime }(\max (1-\xi _{\Delta _{\flat }}^{2}))\big )^{\mho }\right) ^\frac{1}{\mho }}\right) ^{\frac{1}{2}}&\le \left( 1-e^{-\left( \sum _{\flat =1}^{\ell }\Theta _{\flat }\big (-ln^{\backprime }(1-\xi _{\Delta _{\flat }}^{2})\big )^{\mho }\right) ^\frac{1}{\mho }}\right) ^{\frac{1}{2}}\\&\le \left( 1-e^{-\left( \sum _{\flat =1}^{\ell }\Theta _{\flat }\big (-ln^{\backprime }(\min (1-\xi _{\Delta _{\flat }}^{2}))\big )^{\mho }\right) ^\frac{1}{\mho }}\right) ^{\frac{1}{2}}. \end{aligned} \end{aligned}$$

Now, we have

$$\begin{aligned} \left( e^{-\left( \sum _{\flat =1}^{\ell }\Theta _{\flat }\big (-ln^{\backprime }\min \eta _{\Delta _{\flat }}^{2}\big )^{\mho }\right) ^\frac{1}{\mho }}\right) ^{\frac{1}{2}} \le \left( e^{-\left( \sum _{\flat =1}^{\ell }\Theta _{\flat }\big (-ln^{\backprime }\eta _{\Delta _{\flat }}^{2}\big )^{\mho }\right) ^\frac{1}{\mho }}\right) ^{\frac{1}{2}}\le \left( e^{-\left( \sum _{\flat =1}^{\ell }\Theta _{\flat }\big (-ln^{\backprime }\max \eta _{\Delta _{\flat }}^{2}\big )^{\mho }\right) ^\frac{1}{\mho }}\right) ^{\frac{1}{2}}. \end{aligned}$$

Similarly, we have

$$\begin{aligned} \left( e^{-\left( \sum _{\flat =1}^{\ell }\Theta _{\flat }\big (-ln^{\backprime }\min \chi _{\Delta _{\flat }}^{2}\big )^{\mho }\right) ^\frac{1}{\mho }}\right) ^{\frac{1}{2}} \le \left( e^{-\left( \sum _{\flat =1}^{\ell }\Theta _{\flat }\big (-ln^{\backprime }\chi _{\Delta _{\flat }}^{2}\big )^{\mho }\right) ^\frac{1}{\mho }}\right) ^{\frac{1}{2}}\le \left( e^{-\left( \sum _{\flat =1}^{\ell }\Theta _{\flat }\big (-ln^{\backprime }\max \chi _{\Delta _{\flat }}^{2}\big )^{\mho }\right) ^\frac{1}{\mho }}\right) ^{\frac{1}{2}}. \end{aligned}$$

and,

$$\begin{aligned} \left( e^{-\left( \sum _{\flat =1}^{\ell }\Theta _{\flat }\big (-ln^{\backprime }\min \curlyvee _{\Delta _{\flat }}^{2}\big )^{\mho }\right) ^\frac{1}{\mho }}\right) ^{\frac{1}{2}}\le \left( e^{-\left( \sum _{\flat =1}^{\ell }\Theta _{\flat }\big (-ln^{\backprime }\curlyvee _{\Delta _{\flat }}^{2}\big )^{\mho }\right) ^\frac{1}{\mho }}\right) ^{\frac{1}{2}}\le \left( e^{-\left( \sum _{\flat =1}^{\ell }\Theta _{\flat }\big (-ln^{\backprime }\max \curlyvee _{\Delta _{\flat }}^{2}\big )^{\mho }\right) ^\frac{1}{\mho }}\right) ^{\frac{1}{2}}. \end{aligned}$$

Therefore,

$$\begin{aligned} \Delta _{\flat }^{-} \le D-SFAAWA\big (\Delta _{1},\Delta _{2},\ldots ,\Delta _{\ell }\big ) \le \Delta _{\flat }^{+}. \end{aligned}$$

$$\square$$

#### Theorem 4.5

***Monotonicity:***

Assuming $\Delta _{\flat }=\{\xi _{\Delta _{\flat }}, \eta _{\Delta _{\flat }}, \chi _{\Delta _{\flat }}; \curlyvee _{\Delta _{\flat }}\}$, where $\flat =\{1,2,\ldots ,\ell \}$ and $\Delta _{\flat }^{\bigstar }=\{\xi _{\Delta _{\flat }}^{\bigstar }, \eta _{\Delta _{\flat }}^{\bigstar }, \chi _{\Delta _{\flat }}^{\bigstar }; \curlyvee _{\Delta _{\flat }}^{\bigstar }\}$, where $\flat =\{1,2,\ldots ,\ell \}$ represents two sets of D-SFNs. If $\Delta _{\flat } \le \Delta _{\flat }^{\bigstar }$ for $\flat =\{1,2,\ldots ,\ell \}$, then

8 $$\begin{aligned} D-SFAAWA\big (\Delta _{1},\Delta _{2},\ldots ,\Delta _{\ell }\big ) \le D-SFAAWA\big (\Delta _{1}^{\bigstar },\Delta _{2}^{\bigstar },\ldots ,\Delta _{\ell }^{\bigstar }\big ). \end{aligned}$$

#### Proof

The proof of the theorem is simple and straightforward. $\square$

### Exploring D-SFS Aczel–Alsina Geometric Aggregation Operators

In this particular subsection, we have introduced and elucidated the notion of a weighted geometric aggregation operation applied to D-SFSs. This aggregation procedure entails employing the Azcel-Alsina norm, a distinct mathematical framework, to amalgamate information from diverse D-SFSs. The weighted geometric operation takes into account the impact of each element in proportion to assigned weights, offering a nuanced and adaptable approach to data integration within the realm of D-SFSs.

#### Definition 4.6

Consider the collection of D-SFNs denoted as $\Delta _{\flat }=\{\xi _{\Delta _{\flat }}, \eta _{\Delta _{\flat }}, \chi _{\Delta _{\flat }}; \curlyvee _{\Delta _{\flat }}\}$, where $\flat =\{1,2,\ldots ,\ell \}$. A D-SN Azcel-Alsina weighted geometric (D-SFAAWG) AO of dimension $\ell$ is a mapping characterized by a weight vector $\Theta =(\Theta _{1},\Theta _{2},\ldots ,\Theta _{\ell })$, ensuring that $\Theta _{\flat } >0$ and $\sum _{\flat =1}^{\ell } \Theta _{\flat }=1$. The operation is defined as follows:

$$\begin{aligned} D-SFAAWG(\Delta _{1},\Delta _{2},\ldots ,\Delta _{\ell }) = \prod _{\flat =1}^{\ell } \Theta _{\flat } \Delta _{\flat } \end{aligned}$$

#### Theorem 4.7

Assume the collection of D-SFNs as $\Delta _{\flat }=\{\xi _{\Delta _{\flat }}, \eta _{\Delta _{\flat }}, \chi _{\Delta _{\flat }}; \curlyvee _{\Delta _{\flat }}\}$, where $\flat =\{1,2,\ldots ,\ell \}$. A D-SFN Azcel-Alsina weighted geometric (D-SFAAWG) Aggregation Operator of dimension $\ell$ is a mapping characterized by a weight vector $\Theta =(\Theta _{1},\Theta _{2},\ldots ,\Theta _{\ell })$, ensuring that $\Theta _{\flat } >0$ and $\sum _{\flat =1}^{\ell } \Theta _{\flat }=1$ is defined as follows:

$$\begin{aligned}{} & {} D-SFAAWG(\Delta _{1},\Delta _{2},\ldots ,\Delta _{\ell })=\\{} & {} \quad \begin{array}{c} \left( \begin{array}{c} \left( e^{-\left( \sum _{\flat =1}^{\ell }\Theta _{\flat }\big (-ln^{\backprime }\xi _{\Delta _{\flat }}^{2}\big )^{\mho }\right) ^\frac{1}{\mho }}\right) ^{\frac{1}{2}}, \left( e^{-\left( \sum _{\flat =1}^{\ell }\Theta _{\flat }\big (-ln^{\backprime }\eta _{\Delta _{\flat }}^{2}\big )^{\mho }\right) ^\frac{1}{\mho }}\right) ^{\frac{1}{2}},\\ \left( 1-e^{-\left( \sum _{\flat =1}^{\ell }\Theta _{\flat }\big (-ln^{\backprime }(1-\chi _{\Delta _{\flat }}^{2})\big )^{\mho }\right) ^\frac{1}{\mho }}\right) ^{\frac{1}{2}}; \left( e^{-\left( \sum _{\flat =1}^{\ell }\Theta _{\flat }\big (-ln^{\backprime }\curlyvee _{\Delta _{\flat }}^{2}\big )^{\mho }\right) ^\frac{1}{\mho }}\right) ^{\frac{1}{2}} \end{array} \right) \end{array} \end{aligned}$$

It is deducted that the obtained result is again a D-SFN.

#### Proof

The theorem is proven through the application of the induction method, which can be expressed as follows:

First, assume $\ell =2$, we have:

$$\begin{aligned}{} & {} D-SFAAWG(\Delta _{1},\Delta _{2},\ldots ,\Delta _{\ell })= \sum _{\flat =1}^{2}\Theta _{\flat }\Delta _{\flat }\\{} & {} \quad \begin{array}{c} \Theta _{1}\Delta _{1}=\left( \begin{array}{c} \left( e^{-\left( \Theta _{1}\big (-ln^{\backprime }\xi _{\Delta _{1}}^{2}\big )^{\mho }\right) ^\frac{1}{\mho }}\right) ^{\frac{1}{2}}, \left( e^{-\left( \Theta _{1}\big (-ln^{\backprime }\eta _{\Delta _{1}}^{2}\big )^{\mho }\right) ^\frac{1}{\mho }}\right) ^{\frac{1}{2}},\\ \left( 1-e^{-\left( \Theta _{1}\big (-ln^{\backprime }(1-\chi _{\Delta _{1}}^{2})\big )^{\mho }\right) ^\frac{1}{\mho }}\right) ^{\frac{1}{2}}; \left( e^{-\left( \Theta _{1}\big (-ln^{\backprime }\curlyvee _{\Delta _{1}}^{2}\big )^{\mho }\right) ^\frac{1}{\mho }}\right) ^{\frac{1}{2}} \end{array} \right) \end{array}\\{} & {} \quad \begin{array}{c} \Theta _{2}\Delta _{2}=\left( \begin{array}{c} \left( e^{-\left( \Theta _{2}\big (-ln^{\backprime }\xi _{\Delta _{2}}^{2}\big )^{\mho }\right) ^\frac{1}{\mho }}\right) ^{\frac{1}{2}}, \left( e^{-\left( \Theta _{2}\big (-ln^{\backprime }\eta _{\Delta _{2}}^{2}\big )^{\mho }\right) ^\frac{1}{\mho }}\right) ^{\frac{1}{2}},\\ \left( 1-e^{-\left( \Theta _{2}\big (-ln^{\backprime }(1-\chi _{\Delta _{2}}^{2})\big )^{\mho }\right) ^\frac{1}{\mho }}\right) ^{\frac{1}{2}}; \left( e^{-\left( \Theta _{2}\big (-ln^{\backprime }\curlyvee _{\Delta _{2}}^{2}\big )^{\mho }\right) ^\frac{1}{\mho }}\right) ^{\frac{1}{2}} \end{array} \right) \end{array} \end{aligned}$$

Now, applying the defined Aczel–Alsina operation, we have

$$\begin{aligned}{} & {} D-SFAAWG(\Delta _{1},\Delta _{2},\ldots ,\Delta _{\ell })= \Theta _{1}\Delta _{1}\otimes \Theta _{2}\Delta _{2}=\\{} & {} \quad \begin{array}{c} \left( \begin{array}{c} \left( e^{-\left( \Theta _{1}\big (-ln^{\backprime }\xi _{\Delta _{1}}^{2}\big )^{\mho }\right) ^\frac{1}{\mho }}\right) ^{\frac{1}{2}}, \left( e^{-\left( \Theta _{1}\big (-ln^{\backprime }\eta _{\Delta _{1}}^{2}\big )^{\mho }\right) ^\frac{1}{\mho }}\right) ^{\frac{1}{2}},\\ \left( 1-e^{-\left( \Theta _{1}\big (-ln^{\backprime }(1-\chi _{\Delta _{1}}^{2})\big )^{\mho }\right) ^\frac{1}{\mho }}\right) ^{\frac{1}{2}}; \left( e^{-\left( \Theta _{1}\big (-ln^{\backprime }\curlyvee _{\Delta _{1}}^{2}\big )^{\mho }\right) ^\frac{1}{\mho }}\right) ^{\frac{1}{2}} \end{array} \right) \end{array}\\{} & {} \quad \otimes \left( \begin{array}{c} \left( e^{-\left( \Theta _{2}\big (-ln^{\backprime }\xi _{\Delta _{2}}^{2}\big )^{\mho }\right) ^\frac{1}{\mho }}\right) ^{\frac{1}{2}}, \left( e^{-\left( \Theta _{2}\big (-ln^{\backprime }\eta _{\Delta _{2}}^{2}\big )^{\mho }\right) ^\frac{1}{\mho }}\right) ^{\frac{1}{2}},\\ \left( 1-e^{-\left( \Theta _{2}\big (-ln^{\backprime }(1-\chi _{\Delta _{2}}^{2})\big )^{\mho }\right) ^\frac{1}{\mho }}\right) ^{\frac{1}{2}}; \left( e^{-\left( 
\Theta _{2}\big (-ln^{\backprime }\curlyvee _{\Delta _{2}}^{2}\big )^{\mho }\right) ^\frac{1}{\mho }}\right) ^{\frac{1}{2}} \end{array} \right) \\{} & {} \quad \begin{array}{c} =\left( \begin{array}{c} \left( e^{-\left( \Theta _{1}\big (-ln^{\backprime }\xi _{\Delta _{1}}^{2}\big )^{\mho }+\Theta _{2}\big (-ln^{\backprime }\xi _{\Delta _{2}}^{2}\big )^{\mho }\right) ^\frac{1}{\mho }}\right) ^{\frac{1}{2}}, \left( e^{-\left( \Theta _{1}\big (-ln^{\backprime }\eta _{\Delta _{1}}^{2}\big )^{\mho }+\Theta _{2}\big (-ln^{\backprime }\eta _{\Delta _{2}}^{2}\big )^{\mho }\right) ^\frac{1}{\mho }}\right) ^{\frac{1}{2}},\\ \left( 1-e^{-\left( \Theta _{1}\big (-ln^{\backprime }(1-\chi _{\Delta _{1}}^{2})\big )^{\mho }+\Theta _{2}\big (-ln^{\backprime }(1-\chi _{\Delta _{2}}^{2})\big )^{\mho }\right) ^\frac{1}{\mho }}\right) ^{\frac{1}{2}}; \left( e^{-\left( \Theta _{1}\big (-ln^{\backprime }\curlyvee _{\Delta _{1}}^{2}\big )^{\mho }+\Theta _{2}\big (-ln^{\backprime }\curlyvee _{\Delta _{2}}^{2}\big )^{\mho }\right) ^\frac{1}{\mho }}\right) ^{\frac{1}{2}} \end{array} \right) \end{array} \\{} & {} \quad \begin{array}{c} =\left( \begin{array}{c} \left( e^{-\left( \sum _{\flat =1}^{2}\Theta _{\flat }\big (-ln^{\backprime }\xi _{\Delta _{\flat }}^{2}\big )^{\mho }\right) ^\frac{1}{\mho }}\right) ^{\frac{1}{2}}, \left( e^{-\left( \sum _{\flat =1}^{2}\Theta _{\flat }\big (-ln^{\backprime }\eta _{\Delta _{\flat }}^{2}\big )^{\mho }\right) ^\frac{1}{\mho }}\right) ^{\frac{1}{2}},\\ \left( 1-e^{-\left( \sum _{\flat =1}^{2}\Theta _{\flat }\big (-ln^{\backprime }(1-\chi _{\Delta _{\flat }}^{2})\big )^{\mho }\right) ^\frac{1}{\mho }}\right) ^{\frac{1}{2}}; \left( e^{-\left( \sum _{\flat =1}^{2}\Theta _{\flat }\big (-ln^{\backprime }\curlyvee _{\Delta _{\flat }}^{2}\big )^{\mho }\right) ^\frac{1}{\mho }}\right) ^{\frac{1}{2}} \end{array} \right) \end{array} \end{aligned}$$

Thus,the theorem holds true for $\ell =2$.

Now, assume that it is valid for $\ell =w$.

$$\begin{aligned}{} & {} D-SFAAWG(\Delta _{1},\Delta _{2},\ldots ,\Delta _{w})=\\{} & {} \quad \begin{array}{c} \left( \begin{array}{c} \left( e^{-\left( \sum _{\flat =1}^{w}\Theta _{\flat }\big (-ln^{\backprime }\xi _{\Delta _{\flat }}^{2}\big )^{\mho }\right) ^\frac{1}{\mho }}\right) ^{\frac{1}{2}}, \left( e^{-\left( \sum _{\flat =1}^{w}\Theta _{\flat }\big (-ln^{\backprime }\eta _{\Delta _{\flat }}^{2}\big )^{\mho }\right) ^\frac{1}{\mho }}\right) ^{\frac{1}{2}},\\ \left( 1-e^{-\left( \sum _{\flat =1}^{w}\Theta _{\flat }\big (-ln^{\backprime }(1-\chi _{\Delta _{\flat }}^{2})\big )^{\mho }\right) ^\frac{1}{\mho }}\right) ^{\frac{1}{2}}; \left( e^{-\left( \sum _{\flat =1}^{w}\Theta _{\flat }\big (-ln^{\backprime }\curlyvee _{\Delta _{\flat }}^{2}\big )^{\mho }\right) ^\frac{1}{\mho }}\right) ^{\frac{1}{2}} \end{array} \right) \end{array} \end{aligned}$$

Now, we’ll prove that it is valid for $\ell =w+1$.

That is,

$$\begin{aligned}{} & {} D-SFAAWG(\Delta _{1},\Delta _{2},\ldots ,\Delta _{w}, \Delta _{w+1})= \bigg (\sum _{\flat =1}^{w}\Theta _{w}\Delta _{w}\bigg )\otimes \big ( \Theta _{w+1}\Delta _{w+1}\big )= \\{} & {} \quad \begin{array}{c} \left( \begin{array}{c} \left( e^{-\left( \sum _{\flat =1}^{w}\Theta _{\flat }\big (-ln^{\backprime }\xi _{\Delta _{\flat }}^{2}\big )^{\mho }\right) ^\frac{1}{\mho }}\right) ^{\frac{1}{2}}, \left( e^{-\left( \sum _{\flat =1}^{w}\Theta _{\flat }\big (-ln^{\backprime }\eta _{\Delta _{\flat }}^{2}\big )^{\mho }\right) ^\frac{1}{\mho }}\right) ^{\frac{1}{2}},\\ \left( 1-e^{-\left( \sum _{\flat =1}^{w}\Theta _{\flat }\big (-ln^{\backprime }(1-\chi _{\Delta _{\flat }}^{2})\big )^{\mho }\right) ^\frac{1}{\mho }}\right) ^{\frac{1}{2}}; \left( e^{-\left( \sum _{\flat =1}^{w}\Theta _{\flat }\big (-ln^{\backprime }\curlyvee _{\Delta _{\flat }}^{2}\big )^{\mho }\right) ^\frac{1}{\mho }}\right) ^{\frac{1}{2}} \end{array} \right) \end{array}\\{} & {} \quad \otimes \left( \begin{array}{c} \left( e^{-\left( \Theta _{w+1}\big (-ln^{\backprime }\xi _{\Delta _{w+1}}^{2}\big )^{\mho }\right) ^\frac{1}{\mho }}\right) ^{\frac{1}{2}}, \left( e^{-\left( \Theta _{w+1}\big (-ln^{\backprime }\eta _{\Delta _{w+1}}^{2}\big )^{\mho }\right) ^\frac{1}{\mho }}\right) ^{\frac{1}{2}},\\ \left( 1-e^{-\left( \Theta _{w+1}\big (-ln^{\backprime }(1-\chi _{\Delta _{w+1}}^{2})\big )^{\mho }\right) ^\frac{1}{\mho }}\right) ^{\frac{1}{2}}; \left( e^{-\left( \Theta _{w+1}\big (-ln^{\backprime }\curlyvee _{\Delta _{w+1}}^{2}\big )^{\mho }\right) ^\frac{1}{\mho }}\right) ^{\frac{1}{2}} \end{array} \right) \\{} & {} \quad \begin{array}{c} =\left( \begin{array}{c} \left( e^{-\left( \sum _{\flat =1}^{w}\Theta _{\flat }\big (-ln^{\backprime }\xi _{\Delta _{\flat }}^{2}\big )^{\mho }+\Theta _{w+1}\big (-ln^{\backprime }\xi _{\Delta _{w+1}}^{2}\big )^{\mho }\right) ^\frac{1}{\mho }}\right) ^{\frac{1}{2}},\\ \left( e^{-\left( \sum _{\flat =1}^{w}\Theta _{\flat }\big (-ln^{\backprime }\eta _{\Delta _{\flat }}^{2}\big )^{\mho }+\Theta _{w+1}\big (-ln^{\backprime }\eta _{\Delta _{w+1}}^{2}\big )^{\mho }\right) ^\frac{1}{\mho }}\right) ^{\frac{1}{2}},\\ \left( 1-e^{-\left( \sum _{\flat =1}^{w}\Theta _{\flat }\big (-ln^{\backprime }(1-\chi _{\Delta _{\flat }}^{2})\big )^{\mho }+\Theta _{w+1}\big (-ln^{\backprime }(1-\chi _{\Delta _{w+1}}^{2})\big )^{\mho }\right) ^\frac{1}{\mho }}\right) ^{\frac{1}{2}};\\ \left( e^{-\left( \sum _{\flat =1}^{w}\Theta _{\flat }\big (-ln^{\backprime }\curlyvee _{\Delta _{\flat }}^{2}\big )^{\mho }+\Theta _{w+1}\big (-ln^{\backprime }\curlyvee _{\Delta _{w+1}}^{2}\big )^{\mho }\right) ^\frac{1}{\mho }}\right) ^{\frac{1}{2}} \end{array} \right) \end{array}\\{} & {} \quad D-SFAAWG(\Delta _{1},\Delta _{2},\ldots ,\Delta _{w+1})=\\{} & {} \quad \begin{array}{c} \left( \begin{array}{c} \left( e^{-\left( \sum _{\flat =1}^{w+1}\Theta _{\flat }\big (-ln^{\backprime }\xi _{\Delta _{\flat }}^{2}\big )^{\mho }\right) ^\frac{1}{\mho }}\right) ^{\frac{1}{2}}, \left( e^{-\left( \sum _{\flat =1}^{w+1}\Theta _{\flat }\big (-ln^{\backprime }\eta _{\Delta _{\flat }}^{2}\big )^{\mho }\right) ^\frac{1}{\mho }}\right) ^{\frac{1}{2}},\\ \left( 1-e^{-\left( \sum _{\flat =1}^{w+1}\Theta _{\flat }\big (-ln^{\backprime }(1-\chi _{\Delta _{\flat }}^{2})\big )^{\mho }\right) ^\frac{1}{\mho }}\right) ^{\frac{1}{2}}; \left( e^{-\left( \sum _{\flat =1}^{w+1}\Theta _{\flat }\big (-ln^{\backprime }\curlyvee _{\Delta _{\flat }}^{2}\big )^{\mho }\right) ^\frac{1}{\mho }}\right) ^{\frac{1}{2}} \end{array} \right) \end{array} \end{aligned}$$

Hence, the statement of the theorem has been demonstrated to hold true for every possible value of $\ell$, confirming its validity across the entire range of applicable conditions. The proof process undertaken establishes the general applicability and correctness of the theorem, providing a comprehensive and conclusive verification. $\square$

Leveraging the D-SFAAWG operator enables us to efficiently highlight the distinctive characteristics outlined below.

#### Theorem 4.8

***Idempotency:***

Assuming $\Delta _{\flat }=\{\xi _{\Delta _{\flat }}, \eta _{\Delta _{\flat }}, \chi _{\Delta _{\flat }}; \curlyvee _{\Delta _{\flat }}\}$, where $\flat =\{1,2,\ldots ,\ell \}$ represents a set of equivalent D-SFNs, and $\Delta _{\flat }=\Delta$ holds for each $\flat =\{1,2,\ldots ,\ell \}$, then

9 $$\begin{aligned} D-SFAAWG\big (\Delta _{1},\Delta _{2},\ldots ,\Delta _{\ell }\big )=\Delta \end{aligned}$$

#### Proof

Since,

$$\begin{aligned}{} & {} D-SFAAWG(\Delta _{1},\Delta _{2},\ldots ,\Delta _{\ell })=\\{} & {} \quad \begin{array}{c} \left( \begin{array}{c} \left( e^{-\left( \sum _{\flat =1}^{\ell }\Theta _{\flat }\big (-ln^{\backprime }\xi _{\Delta _{\flat }}^{2}\big )^{\mho }\right) ^\frac{1}{\mho }}\right) ^{\frac{1}{2}}, \left( e^{-\left( \sum _{\flat =1}^{\ell }\Theta _{\flat }\big (-ln^{\backprime }\eta _{\Delta _{\flat }}^{2}\big )^{\mho }\right) ^\frac{1}{\mho }}\right) ^{\frac{1}{2}},\\ \left( 1-e^{-\left( \sum _{\flat =1}^{\ell }\Theta _{\flat }\big (-ln^{\backprime }(1-\chi _{\Delta _{\flat }}^{2})\big )^{\mho }\right) ^\frac{1}{\mho }}\right) ^{\frac{1}{2}}; \left( e^{-\left( \sum _{\flat =1}^{\ell }\Theta _{\flat }\big (-ln^{\backprime }\curlyvee _{\Delta _{\flat }}^{2}\big )^{\mho }\right) ^\frac{1}{\mho }}\right) ^{\frac{1}{2}} \end{array} \right) \end{array} \end{aligned}$$

Now, put $\Delta _{\flat }={\xi _{\Delta _{\flat }}, \eta _{\Delta _{\flat }}, \chi _{\Delta _{\flat }}; \curlyvee _{\Delta _{\flat }}}=\Delta$ where $\flat =\{1,2,\ldots ,\ell \}$.

$$\begin{aligned}{} & {} \begin{array}{c} D-SFAAWG(\Delta _{1},\Delta _{2},\ldots ,\Delta _{\ell })\left( \begin{array}{c} \left( e^{-\left( \sum _{\flat =1}^{\ell }\Theta _{\flat }\big (-ln^{\backprime }\xi _{\Delta }^{2}\big )^{\mho }\right) ^\frac{1}{\mho }}\right) ^{\frac{1}{2}}, \left( e^{-\left( \sum _{\flat =1}^{\ell }\Theta _{\flat }\big (-ln^{\backprime }\eta _{\Delta }^{2}\big )^{\mho }\right) ^\frac{1}{\mho }}\right) ^{\frac{1}{2}},\\ \left( 1-e^{-\left( \sum _{\flat =1}^{\ell }\Theta _{\flat }\big (-ln^{\backprime }(1-\chi _{\Delta }^{2})\big )^{\mho }\right) ^\frac{1}{\mho }}\right) ^{\frac{1}{2}}; \left( e^{-\left( \sum _{\flat =1}^{\ell }\Theta _{\flat }\big (-ln^{\backprime }\curlyvee _{\Delta }^{2}\big )^{\mho }\right) ^\frac{1}{\mho }}\right) ^{\frac{1}{2}} \end{array} \right) \end{array}\\{} & {} \quad \begin{array}{c} =\left( \begin{array}{c} \left( e^{-\left( \Theta _{\flat }\big (-ln^{\backprime }\xi _{\Delta }^{2}\big )^{\mho }\right) ^\frac{1}{\mho }}\right) ^{\frac{1}{2}}, \left( e^{-\left( \Theta _{\flat }\big (-ln^{\backprime }\eta _{\Delta }^{2}\big )^{\mho }\right) ^\frac{1}{\mho }}\right) ^{\frac{1}{2}},\\ \left( 1-e^{-\left( \Theta _{\flat }\big (-ln^{\backprime }(1-\chi _{\Delta }^{2})\big )^{\mho }\right) ^\frac{1}{\mho }}\right) ^{\frac{1}{2}}; \left( e^{-\left( \Theta _{\flat }\big (-ln^{\backprime }\curlyvee _{\Delta }^{2}\big )^{\mho }\right) ^\frac{1}{\mho }}\right) ^{\frac{1}{2}} \end{array} \right) \end{array}\\{} & {} \quad \{\xi _{\Delta _{\flat }}, \eta _{\Delta _{\flat }}, \chi _{\Delta _{\flat }}; \curlyvee _{\Delta _{\flat }}\}=\Delta . \end{aligned}$$

Thus, $$D-SFAAWG\big (\Delta _{1},\Delta _{2},\ldots ,\Delta _{\ell }\big )=\Delta$$ holds. $$\square$$

#### Theorem 4.9

***Boundedness:***

Assuming $\Delta _{\flat }=\{\xi _{\Delta _{\flat }}, \eta _{\Delta _{\flat }}, \chi _{\Delta _{\flat }}; \curlyvee _{\Delta _{\flat }}\}$, where $\flat =\{1,2,\ldots ,\ell \}$ represents a set of equivalent D-SFNs. Consider

$$\begin{aligned} \Delta _{\flat }^{+}=\bigg (\max _{\flat }\big \{\xi _{\Delta _{\flat }}\big \}, \min _{\flat }\big \{\eta _{\Delta _{\flat }}\big \}, \min _{\flat }\big \{\chi _{\Delta _{\flat }}\big \}; \min _{\flat }\big \{\curlyvee _{\Delta _{\flat }}\big \}\bigg ) \end{aligned}$$

and

$$\begin{aligned} \Delta _{\flat }^{-}=\bigg (\min _{\flat }\big \{\xi _{\Delta _{\flat }}\big \}, \min _{\flat }\big \{\eta _{\Delta _{\flat }}\big \}, \max _{\flat }\big \{\chi _{\Delta _{\flat }}\big \}; \min _{\flat }\big \{\curlyvee _{\Delta _{\flat }}\big \}\bigg ). \end{aligned}$$

Then,

10 $$\begin{aligned} \Delta _{\flat }^{-} \le D-SFAAWG\big (\Delta _{1},\Delta _{2},\ldots ,\Delta _{\ell }\big ) \le \Delta _{\flat }^{+}. \end{aligned}$$

#### Proof

Considering $\min _{\flat }\big \{\xi _{\Delta _{\flat }}\big \} \le \xi _{\Delta _{\flat }} \le \max _{\flat }\big \{\xi _{\Delta _{\flat }}\big \}$ it can be deduced that:

$$\begin{aligned} \begin{aligned} \left( e^{-\left( \sum _{\flat =1}^{\ell }\Theta _{\flat }\big (-ln^{\backprime }\min \xi _{\Delta _{\flat }}^{2}\big )^{\mho }\right) ^\frac{1}{\mho }}\right) ^{\frac{1}{2}} \left( \le e^{-\left( \sum _{\flat =1}^{\ell }\Theta _{\flat }\big (-ln^{\backprime }\xi _{\Delta _{\flat }}^{2}\big )^{\mho }\right) ^\frac{1}{\mho }}\right) ^{\frac{1}{2}}\le \\ \left( e^{-\left( \sum _{\flat =1}^{\ell }\Theta _{\flat }\big (-ln^{\backprime }\max \xi _{\Delta _{\flat }}^{2}\big )^{\mho }\right) ^\frac{1}{\mho }}\right) ^{\frac{1}{2}}. \end{aligned} \end{aligned}$$

Similarly,

$$\begin{aligned} \left( e^{-\left( \sum _{\flat =1}^{\ell }\Theta _{\flat }\big (-ln^{\backprime }\min \eta _{\Delta _{\flat }}^{2}\big )^{\mho }\right) ^\frac{1}{\mho }}\right) ^{\frac{1}{2}} \le \left( e^{-\left( \sum _{\flat =1}^{\ell }\Theta _{\flat }\big (-ln^{\backprime }\eta _{\Delta _{\flat }}^{2}\big )^{\mho }\right) ^\frac{1}{\mho }}\right) ^{\frac{1}{2}}\le \left( e^{-\left( \sum _{\flat =1}^{\ell }\Theta _{\flat }\big (-ln^{\backprime }\max \eta _{\Delta _{\flat }}^{2}\big )^{\mho }\right) ^\frac{1}{\mho }}\right) ^{\frac{1}{2}}. \end{aligned}$$

Now, we have

$$\begin{aligned} \begin{aligned} \left( 1-e^{-\left( \sum _{\flat =1}^{\ell }\Theta _{\flat }\big (-ln^{\backprime }(\max (1-\chi _{\Delta _{\flat }}^{2}))\big )^{\mho }\right) ^\frac{1}{\mho }}\right) ^{\frac{1}{2}} \le \left( 1-e^{-\left( \sum _{\flat =1}^{\ell }\Theta _{\flat }\big (-ln^{\backprime }(1-\chi _{\Delta _{\flat }}^{2})\big )^{\mho }\right) ^\frac{1}{\mho }}\right) ^{\frac{1}{2}}\le \\ \left( 1-e^{-\left( \sum _{\flat =1}^{\ell }\Theta _{\flat }\big (-ln^{\backprime }(\min (1-\chi _{\Delta _{\flat }}^{2}))\big )^{\mho }\right) ^\frac{1}{\mho }}\right) ^{\frac{1}{2}}. \end{aligned}\end{aligned}$$

and,

$$\begin{aligned} \left( e^{-\left( \sum _{\flat =1}^{\ell }\Theta _{\flat }\big (-ln^{\backprime }\min \curlyvee _{\Delta _{\flat }}^{2}\big )^{\mho }\right) ^\frac{1}{\mho }}\right) ^{\frac{1}{2}}\le \left( e^{-\left( \sum _{\flat =1}^{\ell }\Theta _{\flat }\big (-ln^{\backprime }\curlyvee _{\Delta _{\flat }}^{2}\big )^{\mho }\right) ^\frac{1}{\mho }}\right) ^{\frac{1}{2}}\le \left( e^{-\left( \sum _{\flat =1}^{\ell }\Theta _{\flat }\big (-ln^{\backprime }\max \curlyvee _{\Delta _{\flat }}^{2}\big )^{\mho }\right) ^\frac{1}{\mho }}\right) ^{\frac{1}{2}}. \end{aligned}$$

Therefore,

$$\begin{aligned} \Delta _{\flat }^{-} \le D-SFAAWG\big (\Delta _{1},\Delta _{2},\ldots ,\Delta _{\ell }\big ) \le \Delta _{\flat }^{+}. \end{aligned}$$

$$\square$$

#### Theorem 4.10

***Monotonicity:***

Assuming $\Delta _{\flat }=\{\xi _{\Delta _{\flat }}, \eta _{\Delta _{\flat }}, \chi _{\Delta _{\flat }}; \curlyvee _{\Delta _{\flat }}\}$, where $\flat =\{1,2,\ldots ,\ell \}$ and $\Delta _{\flat }^{\bigstar }=\{\xi _{\Delta _{\flat }}^{\bigstar }, \eta _{\Delta _{\flat }}^{\bigstar }, \chi _{\Delta _{\flat }}^{\bigstar }; \curlyvee _{\Delta _{\flat }}^{\bigstar }\}$, where $\flat =\{1,2,\ldots ,\ell \}$ represents two sets of D-SFNs. If $\Delta _{\flat } \le \Delta _{\flat }^{\bigstar }$ for $\flat =\{1,2,\ldots ,\ell \}$, then

11 $$\begin{aligned} D-SFAAWG\big (\Delta _{1},\Delta _{2},\ldots ,\Delta _{\ell }\big ) \le D-SFAAWG\big (\Delta _{1}^{\bigstar },\Delta _{2}^{\bigstar },\ldots ,\Delta _{\ell }^{\bigstar }\big ). \end{aligned}$$

#### Proof

The proof of the theorem is simple and straightforward. $\square$

## D-SF decision matrix construction

Consider a set of $\ell$ alternatives, each rated based on attributes $\{\gimel _{1},\gimel _{2},\ldots ,\gimel _{\rho }\}$. The significance of individual attributes $\gimel _{\jmath }$, where $(\jmath =1,2,\ldots ,\rho )$ is determined by a vector $\Theta =\{\Theta _{1},\Theta _{2},..,\Theta _{\rho }\}^{T}$, with $\Theta _{\jmath }>0$ and $\sum _{\jmath =1}^{\rho }=1$.

Suppose $\Delta _{\flat _{\jmath }}=\{\xi _{\Delta _{\flat _{\jmath }}},\eta _{\Delta _{\flat _{\jmath }}},\chi _{\Delta _{\flat _{\jmath }}};\curlyvee _{\Delta _{\flat _{\jmath }}}\}$ for each attribute, where $\xi _{\Delta _{\flat _{\jmath }}},\eta _{\Delta _{\flat _{\jmath }}},\chi _{\Delta _{\flat _{\jmath }}};\curlyvee _{\Delta _{\flat _{\jmath }}} \in [0,1]$ represent the acceptable ratings of attributes for each alternative. Here, $\xi _{\Delta _{\flat _{\jmath }}}$ corresponds to the positive grade, $\chi _{\Delta _{\flat _{\jmath }}}$ to the negative grade, $\eta _{\Delta _{\flat _{\jmath }}}$ to abstention, and $\curlyvee _{\Delta _{\flat _{\jmath }}}$ to the radius. The decision matrix obtained from the assessment data is denoted as $\Delta ^{\diamond }=\{\Delta ^{\diamond }_{\flat _{\jmath }}\}_{\ell \rho }$, where $(\diamond =1,2,\ldots ,e.)$.

Following are the steps of planned algorithm:

###  Algorithm 5.1. Initialization of MCDM Algorithm: By Evaluating D-SF-MEREC-SWARA-MARCOS Method

Step I: *Normalize the DM. The normalized matrix elements, denoted as* $$\Delta _{\mathbb {N}} = \{\Delta ^{\centerdot }\}_{\ell \rho }$$, *are differentiated based on* $$\pounds _{\breve{b}}$$ *for benefit criteria and* $$\pounds _{\breve{c}}$$ *for cost criteria.*
$$\begin{aligned} \Delta ^{\centerdot }_{\imath \jmath }=\{\xi ^{\centerdot }_{\imath \jmath },\eta ^{\centerdot }_{\imath \jmath },\chi ^{\centerdot }_{\imath \jmath };\curlyvee ^{\centerdot }_{ \imath \jmath }\}=\left\{ \begin{array}{ll} \Delta ^{\star }_{\imath \jmath }=\{\xi ^{\star }_{\imath \jmath },\eta ^{\star }_{\imath \jmath },\chi ^{\star }_{\imath \jmath };\curlyvee ^{\star }_{\imath \jmath }\}, &{}~ {\text { if}} ~ \rho \in \pounds _{\breve{b}},\\ \big (\Delta ^{\star }_{\imath \jmath }\big )^{c}=\{\chi ^{\star }_{\imath \jmath },\eta ^{\star }_{\imath \jmath },\xi ^{\star }_{\imath \jmath };\curlyvee ^{\star }_{\ell \rho }\}, &{}~ {\text {if}} ~\rho \in \pounds _{\breve{c}}. \end{array} \right. \end{aligned}$$
Step II: *Determine the weights for the DEs* $$\Xi$$.
  $$\begin{aligned} \Xi _{\diamond }=\frac{1+\frac{1}{\diamond }\sum _{e=1}^{\diamond } \big (\xi _{\diamond } log\big (\xi _{\diamond }\big )+\eta _{\diamond } log\big (\eta _{\diamond }\big )+\chi _{\diamond } log\big (\chi _{\diamond }\big )\big )}{\sum _{e=1}^{\diamond }\bigg (1+\frac{1}{\diamond }\sum _{e=1}^{\diamond } \big (\xi _{\diamond } log\big (\xi _{\diamond }\big )+\eta _{\diamond } log\big (\eta _{\diamond }\big )+\chi _{\diamond } log\big (\chi _{\diamond }\big )\big )\bigg )} \end{aligned}$$                                  (12)
  *Here*, $$\Xi _{\diamond }\ge 0$$ and $$\sum _{e=1}^{\diamond }\Xi _{\diamond }=1.$$
Step III: *Specify the Aggregated D-SF Decision Matrix (ADSF-DM) in accordance with the assigned weight by an expert. Assume* $$\Delta =\{\Delta ^{\star }_{\flat _{\rho }}\}_{\ell \rho }$$ *be the ADSF-DM, where*
  $$\begin{aligned}\{\Delta 
^{\star }_{\flat _{\rho }}\}_{\ell \rho }=\Bigg \{ \left( 1-e^{-\left( \sum _{\flat =1}^{\ell }\Xi _{\flat }\big (-ln^{\backprime }(1-\xi _{\Delta _{\flat }}^{2})\big )^{\mho }\right) ^\frac{1}{\mho }}\right) ^{\frac{1}{2}}, \left( e^{-\left( \sum _{\flat =1}^{\ell }\Xi _{\flat }\big (-ln^{\backprime }\eta _{\Delta _{\flat }}^{2}\big )^{\mho }\right) ^\frac{1}{\mho }}\right) ^{\frac{1}{2}},\\ \left( e^{-\left( \sum _{\flat =1}^{\ell }\Xi _{\flat }\big (-ln^{\backprime }\chi _{\Delta _{\flat }}^{2}\big )^{\mho }\right) ^\frac{1}{\mho }}\right) ^{\frac{1}{2}}; \left( e^{-\left( \sum _{\flat =1}^{\ell }\Xi _{\flat }\big (-ln^{\backprime }\curlyvee _{\Delta _{\flat }}^{2}\big )^{\mho }\right) ^\frac{1}{\mho }}\right) ^{\frac{1}{2}} \Bigg \} \end{aligned}$$                                                 (13)
Step IV: *Establish weights for the criteria.*
Step IV(a): *First, assess the Objective Weights (OWs) through the MEREC method*.
  1. *Compute the comprehensive performance of the alternatives* $$\nu _{\imath }$$. *Employ a logarithmic measure with uniformly assigned criteria weights to assess the overall performance in this stage*.
    $$\nu _{\imath }=ln\left( 1+\left( \frac{1}{\rho }\sum _{\jmath =1}^{\rho }\left| ln\left( \Delta ^{\centerdot }_{\imath \jmath }\right) \right| \right) \right) .$$                                  (14)
  2. *Evaluate the performance of alternatives by iteratively excluding each criterion. Utilize the logarithmic function introduced in previous step , with the unique feature being the computation of alternative assessments by removing each criterion individually at this stage. Consequently, n sets of evaluations corresponding to n criteria are obtained. Let* $$\nu _{\imath \jmath }$$ *denote the comprehensive evaluation of the* $$\imath$$*th alternative after eliminating the* $$\jmath$$*th criterion. The assessment process adheres to following equation:*
    $$\nu _{\imath \jmath }^{\prime }=ln\left( 1+\left( \frac{1}{\rho }\sum _{\breve{k}, \breve{k} \ne \jmath }^{\rho }\left| ln\left( \Delta ^{\centerdot }_{\imath \breve{k}}\right) \right| \right) \right) .$$                                  (15)
  3. *Compute the sum of absolute deviations using Equations (9) and (10).*
    $$\Lambda _{\jmath }=\sum _{\imath } \left| \nu _{\imath \jmath }- \nu _{\imath }\right| .$$                                              (16)
  4. *Determine the ultimate OWs for the criteria*
    $$\Theta ^{\bullet }_{\jmath }= \frac{\Lambda _{\jmath }}{\sum _{\jmath =1}^{\rho }\Lambda _{\jmath }}$$.                                             (17)
Step IV(b): *Assess the Subjective Criteria Weights (SCWs) through the SWARA method. The process for evaluating criteria weights using the SWARA technique is outlined as follows:*
  1. *Determine the crisp values by calculating the initial score values* $$\wp \left( \Delta ^{\centerdot }_{\breve{k} \jmath }\right)$$ *of D-SFNs using Equation (5).*
  2. *Calculate the rank of criteria by arranging them in order of significance based on expert insights, from the most to the least significant.*
  3. *Analyze the relative significance* $$\sigma _{\jmath }$$ *of the average value. Determine the relative position from criteria placed at the second position, and subsequent relative importance is derived by comparing criteria at the* $$\jmath$$*th and* $$(\jmath -1)$$*th places.*
  4. *Estimate the relative coefficient* $$\zeta _{\jmath }$$ *by using equation given below.*
$$\begin{aligned} \zeta _{\jmath }=\left\{ \begin{array}{ll} 1, &{}~ {\text { if}} ~ \jmath =1,\\ \sigma _{\jmath }+1, &{}~ {\text {if}} ~\jmath >1. \end{array} \right. \end{aligned}$$
    *In this context*, $$\zeta _{\jmath }$$ *represents the relative significance*.
  5. *Moving on, calculate the recalculated weights* $$(\varphi _{\jmath })$$ *given in equation below.*
$$\begin{aligned} \varphi _{\jmath }=\left\{ \begin{array}{ll} 1, &{}~ {\text { if}} ~ \jmath =1,\\ \frac{\zeta _{\jmath -1}}{\zeta _{\jmath }}, &{}~ {\text {if}} ~\jmath >1. \end{array} \right. \end{aligned}$$
  6. *Evaluate the specified subjective weight as:*
    $$\Theta ^{\textbf{s}}_{\jmath }= \frac{\zeta _{\jmath }}{\sum _{\jmath =1}^{\rho }\zeta _{\jmath }}.$$                                              (18)
Step IV(c): *In the MCDM technique, each criterion holds distinct degrees of significance. Let* $$\Theta =\{\Theta _{1},\Theta _{2},..,\Theta _{\rho }\}^{T}$$, *be the criteria weights, where with* $$\sum _{\rho =1}^{\jmath }=1$$ *and* $$\Theta _{\rho } \in [0, 1]$$. *It is expressed as:*
  $$\Theta _{\jmath }= \frac{\Theta ^{\bullet }_{\jmath }*\Theta ^{\textbf{s}}_{\jmath } }{\sum _{\jmath =1}^{\rho } \Theta ^{\bullet }_{\jmath } *\Theta ^{\textbf{s}}_{\jmath }}.$$                                              (19)
Step V: *Rank the alternatives using the MARCOS method. Assess an expanded initial D-SFDM by computing the D-SF Positive Ideal Solution (D-SF-PIS) & Negative Ideal Solution (PF-NIS).*
$$\nabla ^{+} (PIS)=\max _{\rho }\Delta _{\imath \jmath }^{\star } ~\text {if}~ \rho \in \pounds _{\breve{b}}~~ \& ~~ \nabla ^{+} (PIS)=\min _{\rho }\Delta _{\imath \jmath }^{\star } ~\text {if}~ \rho \in \pounds _{\breve{c}}.$$
$$\nabla ^{-} (NIS)=\min _{\rho }\Delta _{\imath \jmath }^{\star } ~\text {if}~ \rho \in \pounds _{\breve{b}} ~~ \& ~~ \nabla ^{-} (NIS)=\max _{\rho }\Delta _{\imath \jmath }^{\star } ~\text {if}~ \rho \in \pounds _{\breve{c}}.$$
  *where* $$\max _{\rho }\Delta _{\imath \jmath }^{\star }= \{\max _{\rho }\xi _{\imath \jmath },\min _{\rho }\eta _{\imath \jmath },\min _{\rho }\chi _{\imath \jmath };\max _{\rho }\curlyvee _{ \imath \jmath }\}$$    &  
  $$\min _{\rho }\Delta _{\imath \jmath }^{\star }= \{\min _{\rho }\xi _{\imath \jmath },\min _{\rho }\eta _{\imath \jmath },\max _{\rho }\chi _{\imath \jmath };\min _{\rho }\curlyvee _{ \imath \jmath }\}$$.
Step VI: *Calculate the crisp values using Equation (5).*
Step VII: *Normalize the D-SFDM* $$\Delta _{\mathbb {N}}=\left( \Delta ^{\blacklozenge }_{\imath \jmath }\right) _{\ell \rho }$$ *where*,
  $$\begin{aligned} \Delta ^{\blacklozenge }_{\imath \jmath }=\frac{\nabla ^{+}_{\imath \jmath }}{\nabla _{\imath \jmath }} {\text {if}} ~\rho \in \pounds _{\breve{c}}\\ \Delta ^{\blacklozenge }_{\imath \jmath }=\frac{\nabla _{\imath \jmath }}{\nabla ^{+}_{\imath \jmath }} if ~\rho \in \pounds _{\breve{b}} \end{aligned}$$             (20)
Step VIII: *Form the weighted matrix according to equation below, where* $$\Theta _{\jmath }$$ *signifies the combined criterion weights*.
  $$\Re _{\imath \jmath }=\Delta ^{\blacklozenge }_{\imath \jmath } \times \Theta _{\jmath }$$                                              (21)
Step IX: *Calculate the utility degree of the alternatives as follows:*
  $$\yen _{\imath }^{+}=\frac{\mathcal {S_{\imath }}}{\mathcal {S^{+}}} ~ \& ~ \yen _{\imath }^{-}=\frac{\mathcal {S_{\imath }}}{\mathcal {S^{-}}}$$                                              (22)
  *where*, $$\mathcal {S_{\imath }}=\sum _{\jmath =1}^{\rho }\Re _{\imath \jmath }$$.
Step X: *Compute the Utility Function (UF) in relation to the PIS* $$\digamma \left( \yen ^{+}\right)$$ *and* NIS $$\digamma \left( \yen ^{-}\right)$$ *using the following Equations:*
  $$\begin{aligned} \digamma \left( \yen ^{+}\right) =\frac{\yen _{\imath }^{-}}{\yen _{\imath }^{+}+\yen _{\imath }^{-}},\\ \digamma \left( \yen ^{-}\right) =\frac{\yen _{\imath }^{+}}{\yen _{\imath }^{+}+\yen _{\imath }^{-}},\\ \digamma \left( \yen \right) =\frac{\yen _{\imath }^{+}+\yen _{\imath }^{-}}{1+\frac{1-\digamma \left( \yen ^{+}\right) }{\digamma \left( \yen ^{+}\right) }+\frac{1-\digamma \left( \yen ^{-}\right) }{\digamma \left( \yen ^{-}\right) }}. \end{aligned}$$                                              (23)
Step XI: *Find the best alternative.*

The proposed algorithm intricately weaves together three distinct methods to optimize the DM process. First, objective weights are determined systematically using the MEREC approach, providing a quantitative basis for the subsequent decision processes. Second, the SWARA technique smoothly incorporates subjective weights, making qualitative considerations possible. This stage collects complex viewpoints that are important in determining the criteria that are used to make decisions. To sum up, the MARCOS technique is the backbone of the ranking process, offering a dependable and efficient way to evaluate and order options.

**Figure 1:**
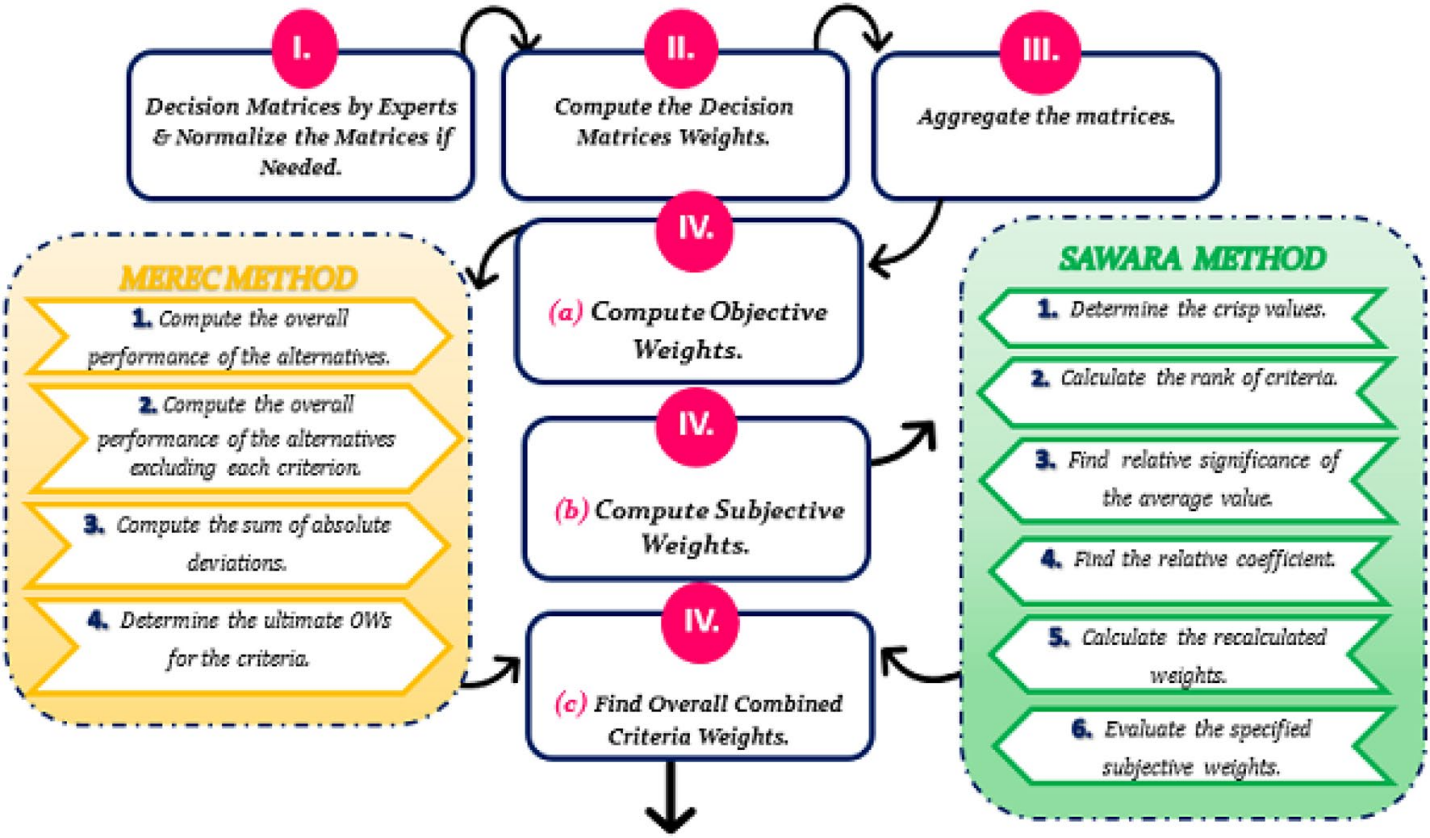
MEREC & SWARAMethods.

**Figure 2:**
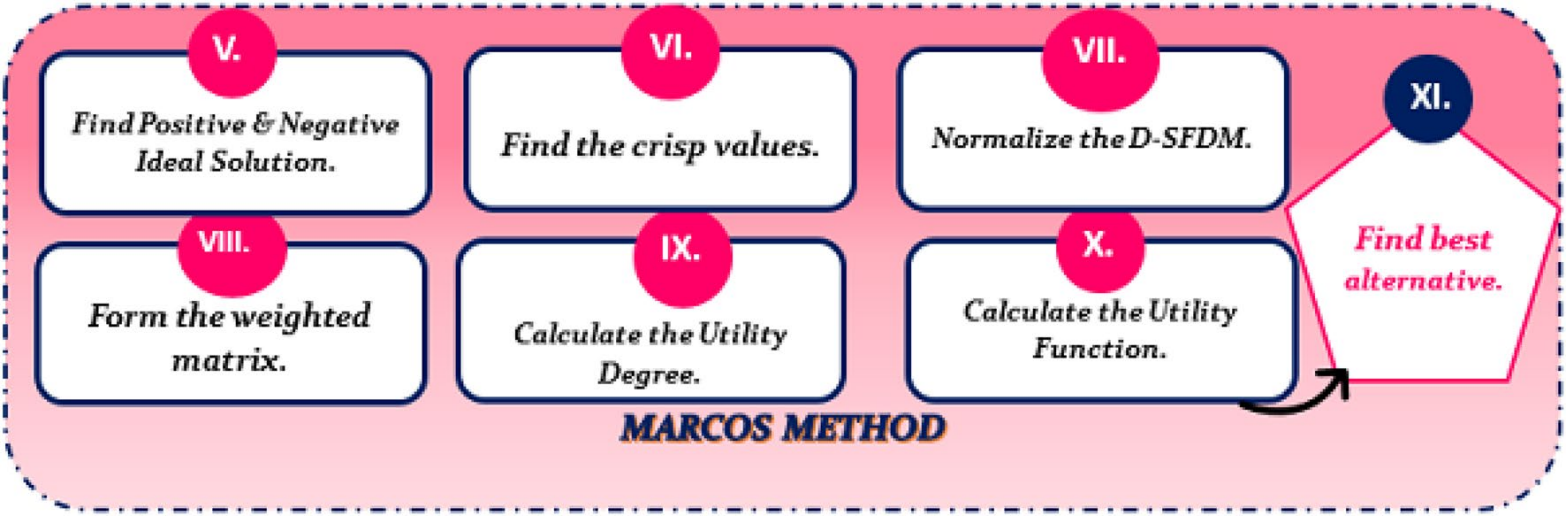
MARCOS Method.

The algorithm becomes readily accessible for comprehension through the aid of the accompanying flowchart in Figures 1 and 2. The graphic depiction acts as a road map, assisting users as they navigate the DM points and sequential stages involved in running the algorithm.

## Case study: advancements in crude oil pretreatment systems

Crude Oil Pretreatment Systems play a pivotal role in the oil refining process, serving as the initial phase dedicated to enhancing the quality and suitability of crude oil for downstream processing. These systems are made to filter contaminants, water, salts, and solid particles out of crude oil, increasing refining efficiency and guaranteeing environmental regulations are met. Emulsion breaking is a common step in the pretreatment process that separates the water and oil, desalting is used to remove salt, dehydration is used to remove water, and filtering is used to remove solid particles. The effective separation of undesired components is further enhanced by chemical treatments and temperature changes. In an effort to achieve greater efficiency and a smaller environmental effect, pretreatment technologies have recently advanced to include novel techniques including membrane filtering and ultrasonic waves. Crude Oil Pretreatment Systems greatly aid in the creation of premium refined goods by utilizing a blend of physical, chemical, and cutting-edge technology procedures. Crude Oil Pretreatment Systems preserve the integrity of downstream equipment and comply with regulatory standards while making a substantial contribution to the manufacture of refined products of superior quality via the use of a mix of physical, chemical, and cutting-edge technical processes.

The Kingdom of Saudi Arabia (KSA) is a key player in the global oil market, which highlights the need for improvements in crude oil pretreatment systems. As the world’s oil consumption grows, it is critical to maximize crude oil’s pretreatment in order to guarantee effective extraction and processing. Improving extracted crude quality is consistent with Saudi Arabia’s objective to fulfill global norms and provide high-quality refined products. Furthermore, environmental stewardship is highly valued in the Kingdom, which makes the use of cutting-edge pretreatment technology necessary to lessen the negative effects of oil production on the environment. The Kingdom of Saudi Arabia (KSA) recognizes the pressing need for improvements Saudi Arabia, a country at the forefront of technical innovation, aspires to implement cutting-edge solutions, and developments in crude oil pretreatment systems are essential to this goal. Effective pretreatment is in line with worldwide initiatives for environmental responsibility and resource optimization in the energy industry, as well as the sustainable control of crude oil’s water content.

In addressing the pressing needs for enhancing crude oil pretreatment, several viable options can be considered to optimize the efficiency and effectiveness of this crucial stage in the oil production process.

1. *Chemical injection system (CIS)*($\Omega _{1}$) An essential part of the oil and gas industry, the Chemical Injection System (CIS) is responsible for refining and quality control of crude oil. In order to improve separation efficiency and lessen the effect of contaminants, this system strategically introduces chemical agents into the crude oil stream. Chemicals that are frequently utilized include demulsifiers, corrosion inhibitors, and scale inhibitors; each has a distinct purpose in the treatment procedure. Demulsifiers help disintegrate emulsions so that impurities and water may be easily separated from crude oil. While scale inhibitors lessen the production of scales that might obstruct effective operations, corrosion inhibitors safeguard the integrity of processing equipment by limiting corrosive reactions. The Chemical Injection System makes sure that crude oil is pretreated more efficiently and effectively, which improves the overall effectiveness, quality, and environmental sustainability of the oil refining operations.
2. *Ultrasonic pretreatment*($\Omega _{2}$) An inventive and effective technique used in the oil and gas sector to improve crude oil separation and processing is ultrasonic pretreatment. With this method, cavitation in the crude oil-water combination is induced using ultrasonic waves, which are usually in the range of 20 kHz to several megahertz. Cavitation causes small bubbles to develop and burst quickly, which breaks up emulsions and makes it easier to separate water and other contaminants from crude oil. Benefits of ultrasonic pretreatment include decreased viscosity, better coalescence, and increased total separation efficiency. Because ultrasonic waves are non-intrusive, this technology is especially appealing because it may be used to many phases of oil production, refining, and wastewater treatment. Ultrasonic pretreatment stands up as a viable method for maximizing crude oil processing while addressing environmental concerns as the oil sector searches for economical and sustainable solutions.
3. *Membrane filtration* ($\Omega _{3}$) Membrane filtration is an essential technology used in many different sectors. It is used to separate and purify gases and liquids. Membrane filtration is a useful technique for the processing of crude oil as it can effectively remove solid particles, water, and contaminants. Semi-permeable membranes, which selectively let some substances to pass through while obstructing others, are used in this procedure. Membrane filtration is an effective technique in the oil and gas industry that helps to produce refined products of superior quality by effectively removing undesirable components from crude oil. Benefits of this technology include its small design, energy economy, and ambient temperature operation. Membrane filtering stands out as an affordable and ecologically responsible way to increase the overall effectiveness of crude oil pretreatment procedures, particularly in light of the oil industry’s growing desire for cleaner and more sustainable methods.
4. *Hybrid pretreatment system* ($\Omega _{4}$) One major development in the field of treating crude oil is the Hybrid Pretreatment System. This cutting-edge system integrates many pretreatment methods to effectively tackle the difficult issues related to removing impurities and improving the quality of crude oil. The Hybrid Pretreatment System combines many processes, including membrane filtration, chemical injection, and electrostatic coalescers, to optimize the process overall and produce better separation efficiency. With its hybrid approach, the KSA’s varied oil reserves have a flexible and adaptable platform that can be modified to particular features of crude oil. The Hybrid Pretreatment System is a viable solution to satisfy urgent demands in the oil industry’s ongoing evolution. It guarantees environmental sustainability and efficiency advantages throughout the crucial pretreatment phase of crude oil.

1. *Separation efficiency*:($\gimel _{1}$) In crude oil pretreatment, separation efficiency describes the system’s capacity to efficiently eliminate impurities, water, and pollutants, guaranteeing that the processed oil satisfies quality requirements. Increased separation efficiency reduces the amount of unwanted components in the final product and increases the total yield of refined goods.
2. *Energy consumption*:($\gimel _{2}$) The quantity of energy needed for procedures like heating, pumping, and separation is known as energy consumption, and it plays a crucial role in the pretreatment of crude oil. By lowering the carbon footprint connected with oil processing, efficient pretreatment systems also help to achieve sustainability goals by minimizing energy use.
3. *Environmental impact*:($\gimel _{3}$) The pretreatment of crude oil has an influence on the environment in terms of emissions, waste production, and overall ecological effects. By combining environmentally friendly techniques, lowering the discharge of pollutants, and cooperating with international initiatives to support sustainable and ethical oil processing, advanced pretreatment technologies aim to lessen their negative effects on the environment.
4. *Scalability and compatibility*:($\gimel _{4}$) Scalability and compatibility describe how well a pretreatment system can adjust to different capacities and work well with already-existing oil processing facilities. While compatibility guarantees seamless interface with other refining processes, ensuring operational flexibility and efficiency, scalability allows a system to adapt to variations in crude oil amounts.

### Numerical example

*Step I*: First, consider the data of D-SF as shown in Table 1. Since there is no criteria in cost form, normalize is skipped.

*Step II*: The D-SFNs presented by three experts include decision matrices, each associated with importance ratings of (0.5, 0.3, 0.6), (0.65, 0.2, 0.3), and (0.7, 0.4, 0.3) for their respective assessments. Determine the weights for the DEs $\Xi$ using equation 12.
  $$\begin{aligned} \Xi _{1}=0.331, \Xi _{2}=0.335,\Xi _{3}= 0.334. \end{aligned}$$
*Step III*: Specify the Aggregated D-SF Decision Matrix (ADSF-DM) in accordance with the assigned weight by an expert using equation 13. The results are presented in Table 2.

*Step IV*: Establish weights for the criteria.
*Step IV(a)*: First, assess the Objective Weights (OWs) through the MEREC method.

1. Compute the comprehensive performance of the alternatives $\nu _{\imath }$ as given in 24.
  24 $$\begin{aligned} \Omega _{1}= 0.179, \Omega _{2}=0.210,\Omega _{3}=0.226, \Omega _{4}=0.180. \end{aligned}$$
2. Evaluate the performance of alternatives by iteratively excluding each criterion as given in Table 3.

3. Compute the sum of absolute deviations as shown in Table 4.
4. Determine the ultimate OWs for the criteria as shown in Table 4.

*Step IV(b)*: Assess the Subjective Criteria Weights (SCWs) through the SWARA method. The process for evaluating criteria weights using the SWARA technique is outlined as follows:

1. Determine the crisp values by calculating the initial score values $\wp \left( \Delta ^{\centerdot }_{\breve{k} \jmath }\right)$ of D-SFNs using Equation (5) as given in Table 5.

2. Calculate the rank of criteria by arranging them in order of significance based on expert insights, from the most to the least significant as given in Table 6.
3. Analyze the relative significance $\sigma _{\jmath }$ of the average value as given in Table 6.
4. Estimate the relative coefficient $\zeta _{\jmath }$ as given in Table 6.
5. Moving on, calculate the recalculated weights $(\varphi _{\jmath })$. The results are shown in Table 6.
6. Evaluate the specified subjective weight as given in Table 6.

*Step IV(c)*: In the MCDM technique, each criterion holds distinct degrees of significance. Let $\Theta =\{\Theta _{1},\Theta _{2},..,\Theta _{\rho }\}^{T}$, be the criteria weights as given in Table 7.

*Step V*: Rank the alternatives using the MARCOS method. Assess an expanded initial D-SFDM by computing the D-SF Positive Ideal Solution (D-SF-PIS) & Negative Ideal Solution (PF-NIS) as given in Table 8.

*Step VI*: Calculate the crisp (score) values a shown in Table 9.

*Step VII*: Normalize the D-SFDM as given in Table 10.

*Step VIII*: Form the weighted matrix according to equation below, where $\Theta _{\jmath }$ signifies the combined criterion weights as in Table 11.

*Step IX*: Calculate the utility degree of the alternatives as in Table 12.
*Step X*: Compute the Utility Function (UF) in relation to the PIS $\digamma \left( \yen ^{+}\right)$ and NIS $\digamma \left( \yen ^{-}\right)$ as given in Table 12.
*Step XI*: Find the best alternative as given in Table 12.

**Table 1:** D-SF Group Decision Matrix.

$\Delta ^{1}$	$\gimel _{1}$	$\gimel _{2}$	$\gimel _{3}$	$\gimel _{4}$
$\Omega _{1}$	$\left( 0.2,0.5,0.3;0.8\right)$	$\left( 0.5,0.4,0.3;0.2\right)$	$\left( 0.4,0.6,0.2;0.1\right)$	$\left( 0.1,0.7,0.3;0.5\right)$
$\Omega _{2}$	$\left( 0.2,0.5,0.4;0.7\right)$	$\left( 0.5,0.4,0.4;0.4\right)$	$\left( 0.4,0.6,0.5;0.3\right)$	$\left( 0.1,0.7,0.5;0.5\right)$
$\Omega _{3}$	$\left( 0.2,0.5,0.5;0.6\right)$	$\left( 0.5,0.4,0.5;0.6\right)$	$\left( 0.4,0.6,0.3;0.5\right)$	$\left( 0.1,0.7,0.7;0.1\right)$
$\Omega _{4}$	$\left( 0.2,0.5,0.6;0.5\right)$	$\left( 0.5,0.4,0.6;0.8\right)$	$\left( 0.4,0.6,0.4;0.7\right)$	$\left( 0.1,0.7,0.2;0.3\right)$

$\Delta ^{2}$	$\gimel _{1}$	$\gimel _{2}$	$\gimel _{3}$	$\gimel _{4}$
$\Omega _{1}$	$\left( 0.3,0.5,0.3;0.2\right)$	$\left( 0.1,0.4,0.3;0.3\right)$	$\left( 0.6,0.6,0.2;0.3\right)$	$\left( 0.2,0.7,0.3;0.2\right)$
$\Omega _{2}$	$\left( 0.3,0.5,0.4;0.1\right)$	$\left( 0.1,0.4,0.4;0.2\right)$	$\left( 0.6,0.5,0.5;0.4\right)$	$\left( 0.3,0.7,0.5;0.3\right)$
$\Omega _{3}$	$\left( 0.3,0.5,0.5;0.4\right)$	$\left( 0.1,0.4,0.5;0.5\right)$	$\left( 0.6,0.5,0.3;0.7\right)$	$\left( 0.4,0.7,0.5;0.3\right)$
$\Omega _{4}$	$\left( 0.3,0.5,0.6;0.5\right)$	$\left( 0.1,0.4,0.6;0.1\right)$	$\left( 0.6,0.4,0.4;0.6\right)$	$\left( 0.1,0.7,0.2;0.7\right)$

$\Delta ^{3}$	$\gimel _{1}$	$\gimel _{2}$	$\gimel _{3}$	$\gimel _{4}$
$\Omega _{1}$	$\left( 0.2,0.4,0.3;0.8\right)$	$\left( 0.5,0.3,0.3;0.1\right)$	$\left( 0.5,0.6,0.2;0.2\right)$	$\left( 0.2,0.7,0.3;0.5\right)$
$\Omega _{2}$	$\left( 0.2,0.5,0.4;0.4\right)$	$\left( 0.3,0.4,0.4;0.4\right)$	$\left( 0.4,0.2,0.5;0.4\right)$	$\left( 0.1,0.4,0.5;0.4\right)$
$\Omega _{3}$	$\left( 0.2,0.4,0.5;0.4\right)$	$\left( 0.2,0.4,0.5;0.1\right)$	$\left( 0.5,0.6,0.3;0.4\right)$	$\left( 0.2,0.7,0.5;0.1\right)$
$\Omega _{4}$	$\left( 0.2,0.5,0.6;0.5\right)$	$\left( 0.5,0.4,0.3;0.8\right)$	$\left( 0.4,0.3,0.4;0.1\right)$	$\left( 0.3,0.7,0.2;0.2\right)$

**Table 2:** Aggregated Matrix.

$\Delta ^{1}$	$\gimel _{1}$	$\gimel _{2}$	$\gimel _{3}$	$\gimel _{4}$
$\Omega _{1}$	$\left( 0.239,0.464,0.300;0.503\right)$	$\left( 0.421,0.363,0.300;0.182\right)$	$\left( 0.511,0.600,0.200;0.182\right)$	$\left( 0.174,0.700,0.300;0.368\right)$
$\Omega _{2}$	$\left( 0.239,0.500,0.400;0.303\right)$	$\left( 0.349,0.400,0.400;0.317\right)$	$\left( 0.483,0.391,0.500;0.364\right)$	$\left( 0.194,0.581,0.500;0.391\right)$
$\Omega _{3}$	$\left( 0.239,0.464,0.500;0.457\right)$	$\left( 0.326,0.400,0.500;0.310\right)$	$\left( 0.511,0.564,0.300;0.519\right)$	$\left( 0.269,0.700,0.559;0.144\right)$
$\Omega _{4}$	$\left( 0.239,0.500,0.600;0.500\right)$	$\left( 0.421,0.400,0.476;0.399\right)$	$\left( 0.483,0.416,0.400;0.347\right)$	$\left( 0.194,0.700,0.200;0.348\right)$

**Table 3:** Comprehensive performance of $\Omega _{\imath }$ by removing each criterion ($\nu _{\imath \jmath }^{\prime }$.).

	$\gimel _{1}$	$\gimel _{2}$	$\gimel _{3}$	$\gimel _{4}$
$\Omega _{1}$	0.18	0.19	0.18	0.15
$\Omega _{2}$	0.19	0.21	0.22	0.18
$\Omega _{3}$	0.22	0.22	0.26	0.16
$\Omega _{4}$	0.16	0.19	0.19	0.16

**Table 4:** Deviation & Weights.

	$\Lambda _{\jmath }$	$\Theta _{\jmath }^{\bullet }$
$\gimel _{1}$	0.0562	0.2039
$\gimel _{2}$	0.0206	0.0748
$\gimel _{3}$	0.0567	0.2054
$\gimel _{4}$	0.1423	0.5159

**Table 5:** Assessment of criteria weights.

	$\Delta ^{1}$	$\Delta ^{2}$	$\Delta ^{3}$	Aggregated D-SFNs
$\Omega _{1}$	$\left( 0.200,0.500,0.436;0.640\right)$	$\left( 0.300,0.500,0.436;0.251\right)$	$\left( 0.200,0.447,0.436;0.503\right)$	$\left( 0.239,0.482,0.436;0.432\right)$
$\Omega _{2}$	$\left( 0.500,0.400,0.436;0.443\right)$	$\left( 0.100,0.400,0.436,0.234\right)$	$\left( 0.403,0.372,0.366;0.238\right)$	$\left( 0.382,0.391,0.411;0.291\right)$
$\Omega _{3}$	$\left( 0.400,0.600,0.331;0.320\right)$	$\left( 0.600,0.495,0.331;0.474\right)$	$\left( 0.454,0.383,0.331;0.238\right)$	$\left( 0.497,0.484,0.331;0.331\right)$
$\Omega _{4}$	$\left( 0.100,0.700,0.381;0.294\right)$	$\left( 0.277,0.609,0.350;0.251\right)$	$\left( 0.213,0.609,0.350;0.251\right)$	$\left( 0.211,0.637,0.360;0.265\right)$

**Table 6:** Calculated SCWs by the D-SF-SWARA method.

Criteria	Score Values	$\sigma _{\jmath }$	$\zeta _{\jmath }$	$\varphi _{\jmath }$	$\Theta ^{\textbf{s}}_{\jmath }$
$\gimel _{3}$	0.124	–	1	1	0.2546
$\gimel _{2}$	0.086	0.038	1.038	0.9634	0.2453
$\gimel _{1}$	0.063	0.023	1.023	1.0147	0.2584
$\gimel _{4}$	− 0.015	0.078	1.078	0.9493	0.2417

**Table 7:** Overall combined weights.

Criterias	$\Theta ^{\bullet }_{\jmath }$	$\Theta ^{\textbf{s}}_{\jmath }$	$\Theta _{\jmath }$
$\gimel _{1}$	0.194	0.258	0.202
$\gimel _{2}$	0.065	0.245	0.065
$\gimel _{3}$	0.205	0.255	0.211
$\gimel _{4}$	0.535	0.242	0.522

**Table 8:** PIS and NIS determined for the D-SF matrix.

$\Delta ^{1}$	$\gimel _{1}$	$\gimel _{2}$	$\gimel _{3}$	$\gimel _{4}$
$\Omega _{1}$	$\left( 0.239,0.464,0.300;0.503\right)$	$\left( 0.421,0.363,0.300;0.182\right)$	$\left( 0.511,0.600,0.200;0.182\right)$	$\left( 0.174,0.700,0.300;0.368\right)$
$\Omega _{2}$	$\left( 0.239,0.500,0.400;0.303\right)$	$\left( 0.349,0.400,0.400;0.317\right)$	$\left( 0.483,0.391,0.500;0.364\right)$	$\left( 0.194,0.581,0.500;0.391\right)$
$\Omega _{3}$	$\left( 0.239,0.464,0.500;0.457\right)$	$\left( 0.326,0.400,0.500;0.310\right)$	$\left( 0.511,0.564,0.300;0.519\right)$	$\left( 0.269,0.700,0.559;0.144\right)$
$\Omega _{4}$	$\left( 0.239,0.500,0.600;0.500\right)$	$\left( 0.421,0.400,0.476;0.399\right)$	$\left( 0.483,0.416,0.400;0.347\right)$	$\left( 0.194,0.700,0.200;0.348\right)$
$\nabla ^{+}$	$\left( 0.239,0.464,0.300;0.503\right)$	$\left( 0.421,0.363,0.300;0.399\right)$	$\left( 0.511,0.391,0.200;0.519\right)$	$\left( 0.269,0.581,0.200;0.391\right)$
$\nabla ^{-}$	$\left( 0.239,0.464,0.600;0.303\right)$	$\left( 0.326,0.363,0.500;0.182\right)$	$\left( 0.483,0.391,0.500;0.182\right)$	$\left( 0.174,0.581,0.559;0.144\right)$

**Table 9:** Score Values.

	$\gimel _{1}$	$\gimel _{2}$	$\gimel _{3}$	$\gimel _{4}$
$\Omega _{1}$	0.12	0.09	0.08	0.01
$\Omega _{2}$	0.03	0.09	0.11	$-$0.001
$\Omega _{3}$	0.06	0.05	0.17	$-$0.11
$\Omega _{4}$	0.03	0.11	0.13	0.03
$\nabla ^{+}$	0.12	0.16	0.23	0.09
$\nabla ^{-}$	− 0.01	0.02	0.05	$-$0.11

**Table 10:** D-SF linear normalized decision matrix.

	$\gimel _{1}$	$\gimel _{2}$	$\gimel _{3}$	$\gimel _{4}$
$\Omega _{1}$	1.000	0.554	0.335	0.075
$\Omega _{2}$	0.245	0.532	0.473	$-$0.007
$\Omega _{3}$	0.484	0.328	0.709	$-$1.211
$\Omega _{4}$	0.291	0.673	0.533	0.342
$\nabla ^{+}$	1.000	1.000	1.000	1.000
$\nabla ^{-}$	$-$0.099	0.101	0.208	$-$1.148

**Table 11:** Weighted Normalized Decision Matrix.

	$\gimel _{1}$	$\gimel _{2}$	$\gimel _{3}$	$\gimel _{4}$
$\Omega _{1}$	0.202	0.036	0.071	0.353
$\Omega _{2}$	0.049	0.034	0.100	$-$0.180
$\Omega _{3}$	0.098	0.021	0.150	$-$0.364
$\Omega _{4}$	0.059	0.043	0.112	0.393
$\nabla ^{+}$	0.202	0.065	0.211	1.000
$\nabla ^{-}$	$-$0.020	0.006	0.044	$-$0.569

**Table 12:** Ranking order of D-SF-MARCOS.

	$\yen ^{+}$	$\yen ^{-}$	$\digamma \left( \yen ^{+}\right)$	$\digamma \left( \yen ^{-}\right)$	$\digamma \left( \yen \right)$	Ranking
$\Omega _{1}$	0.353	$-$0.620	2.321	$-$1.321	0.201	2
$\Omega _{2}$	0.180	$-$0.317	2.321	$-$1.321	0.103	3
$\Omega _{3}$	$-$0.364	0.639	2.321	$-$1.321	$-$0.208	4
$\Omega _{4}$	0.393	$-$0.691	2.321	$-$1.321	0.225	1

## Comparison

This section provides a comparative study of our suggested approach to establish its validity, reliability, and efficacy. There are two components to the comparative research.

In the first section, we present a way for comparing our methods. We use a numerical example solved using our suggested technique as the foundation for this comparison. The ranks are matched appropriately.

In the second section, we use SF information from current literature. We transform this data into our D-SF structure and compare the rankings produced by our proposed approach to those of other aggregation processes in the literature.

### Comparative analysis with a technique

To underscore the robustness of our proposed method in D-SF, we conducted a thorough evaluation by benchmarking it against the well-established CoCoSo method. This comparative analysis serves to enhance the credibility and validity of our approach.

#### Algorithm 7.1. Analyzing D-SF Performance: Insights from a Comparative CoCoSo Perspective

Step 1: *Initiate the analysis with a decision matrix* $\Delta ^{\diamond }=\{\Delta ^{\diamond }_{\flat _{\jmath }}\}_{\ell \rho }$, where $(\diamond =1,2,\ldots ,e.)$, encompassing alternatives $\{\gimel _{1},\gimel _{2},\ldots ,\gimel _{\rho }\}$.
Step 2: *Proceed by normalizing the data, especially for attributes related to costs. The normalized matrix elements, denoted as* $\Delta _{\mathbb {N}} = \{\Delta ^{\centerdot }\}_{\ell \rho }$, *are differentiated based on* $\pounds _{\breve{b}}$ *for benefit criteria and* $\pounds _{\breve{c}}$ *for cost criteria.*
$$\begin{aligned} \Delta ^{\centerdot }_{\imath \jmath }=\{\xi ^{\centerdot }_{\imath \jmath },\eta ^{\centerdot }_{\imath \jmath },\chi ^{\centerdot }_{\imath \jmath };\curlyvee ^{\centerdot }_{ \imath \jmath }\}=\left\{ \begin{array}{ll} \Delta ^{\star }_{\imath \jmath }=\{\xi ^{\star }_{\imath \jmath },\eta ^{\star }_{\imath \jmath },\chi ^{\star }_{\imath \jmath };\curlyvee ^{\star }_{\imath \jmath }\}, &{}~ {\text { if}} ~ \rho \in \pounds _{\breve{b}},\\ \big (\Delta ^{\star }_{\imath \jmath }\big )^{c}=\{\chi ^{\star }_{\imath \jmath },\eta ^{\star }_{\imath \jmath },\xi ^{\star }_{\imath \jmath };\curlyvee ^{\star }_{\ell \rho }\}, &{}~ {\text {if}} ~\rho \in \pounds _{\breve{c}}. \end{array} \right. \end{aligned}$$
Step 3: *Utilize the normalized group decision matrix* $\Delta ^{\centerdot }_{\imath \jmath }$, *and employ the D-SFWA operator to determine the weighted sum measure.*
$$\begin{aligned} D-SFAAWA(\Delta _{1},\Delta _{2},\ldots ,\Delta _{\ell })= \Bigg \{1-e^{-\left( \sum _{\flat =1}^{\ell }\Theta _{\flat }\big (-ln^{\backprime }(1-\xi _{\Delta _{\flat }}^{2})\big )^{\mho }\right) ^\frac{1}{\mho }}, e^{-\left( \sum _{\flat =1}^{\ell }\Theta _{\flat }\big (-ln^{\backprime }\eta _{\Delta _{\flat }}^{2}\big )^{\mho }\right) ^\frac{1}{\mho }},\\ e^{-\left( \sum _{\flat =1}^{\ell }\Theta _{\flat }\big (-ln^{\backprime }\chi _{\Delta _{\flat }}^{2}\big )^{\mho }\right) ^\frac{1}{\mho }}; e^{-\left( \sum _{\flat =1}^{\ell }\Theta _{\flat }\big (-ln^{\backprime }\curlyvee _{\Delta _{\flat }}^{2}\big )^{\mho }\right) ^\frac{1}{\mho }} \Bigg \}. \end{aligned}$$
Step 4: *Utilize the normalized group decision matrix* $\Delta ^{\centerdot }_{\imath \jmath }$, *and employ the D-SFWG operator to determine the weighted sum measure.*
$$\begin{aligned} D-SFAAWG(\Delta _{1},\Delta _{2},\ldots ,\Delta _{\ell })= \Bigg \{e^{-\left( \sum _{\flat =1}^{\ell }\Theta _{\flat }\big (-ln^{\backprime }\xi _{\Delta _{\flat }}\big )^{\mho }\right) ^\frac{1}{\mho }}, e^{-\left( \sum _{\flat =1}^{\ell }\Theta _{\flat }\big (-ln^{\backprime }\eta _{\Delta _{\flat }}^{2}\big )^{\mho }\right) ^\frac{1}{\mho }},\\ 1-e^{-\left( \sum _{\flat =1}^{\ell }\Theta _{\flat }\big (-ln^{\backprime }(1-\chi _{\Delta _{\flat }})\big )^{\mho }\right) ^\frac{1}{\mho }}; e^{-\left( \sum _{\flat =1}^{\ell }\Theta _{\flat }\big (-ln^{\backprime }\curlyvee _{\Delta _{\flat }}^{2}\big )^{\mho }\right) ^\frac{1}{\mho }} \Bigg \}. \end{aligned}$$
Step 5: *Determine the score for both the weighted sum measure denoted as* $\alpha (\Omega _{\imath })$ *and the weighted product measure denoted as* $\beta (\Omega _{\imath })$.
Step 6: *Calculate the relative importance measure of the scheme using three appraisal score strategies.*
  $$\tau _{\imath }^{1}=\frac{\alpha _{\imath }+\beta _{\imath }}{\sum _{\imath =1}^{\ell } (\alpha _{\imath }+\beta _{\imath })},$$                                  (25)
  $$\tau _{\imath }^{2}=\frac{\alpha _{\imath }}{\min _{\imath }\alpha _{\imath }}+\frac{\beta _{\imath }}{\min _{\imath }\beta _{\imath }},$$                                  (26)
  $$\tau _{\imath }^{3}=\frac{\sigma \alpha _{\imath }+(1-\sigma )\beta _{\imath }}{\sigma \max _{\imath }\alpha _{\imath }+(1-\sigma )\max _{\imath }\beta _{\imath }}, \sigma \in [0,1].$$         (27)
Step 7: *The ultimate appraisal index* $\tau _{\imath }$ *was computed by amalgamating the aforementioned three score strategies.*
  $$\tau _{\imath }=\root 3 \of {\tau _{\imath }^{1}\tau _{\imath }^{2}\tau _{\imath }^{3}}+\frac{\tau _{\imath }^{1}+\tau _{\imath }^{2}+\tau _{\imath }^{3}}{3}.$$                                  (28)
Step 8: *Arrange the schemes in decreasing order based on their* $\tau _{\imath }$ *values.*

**Figure 3:**
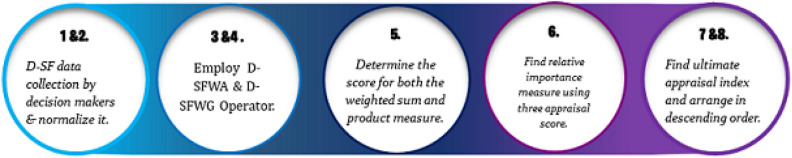
CoCoSo Method.

The visual representation is given in figure 3.

### Numerical example

This section tries to demonstrate the efficiency and reliability of our suggested method in the field of MADM utilizing Combined Compromise Solution (CoCoSo) Method . We will review the case study that was previously covered to demonstrate how our suggested algorithms can skillfully aid in DM by taking a wide range of aspects into account.

*Step 1*: Consider the D-SF group decision matrix and aggregated D-SF information matrix as in Table 1,2.
*Step 2*: Given that there are no cost requirements at play, normalization can be skipped in this situation.
*Step 3,4*: Utilize the 2 we employ the D-SFWA, D-SFWG operator to determine the weighted sum and product measure with criteria weights as $\Theta =\{0.340, 0.230, 0.109, 0.321\}$.
*Step 5*: Determine the score for both the weighted sum measure denoted as $\alpha (\Omega _{\imath })$ and the weighted product measure denoted as $\beta (\Omega _{\imath })$ as given in Table 13.

*Step 6,7*: Calculate the relative importance measure of the scheme using three appraisal score strategies and the ultimate appraisal index $\tau _{\imath }$, as given in Table 14.
*Step 8*: Arrange the schemes in decreasing order based on their $\tau _{\imath }$ values as given in Table 14.

**Table 13:** Score values.

Alternatives	$\alpha (\Omega _{\imath })$	$\beta (\Omega _{\imath })$
$\Omega _{1}$	0.080	0.059
$\Omega _{2}$	0.048	0.077
$\Omega _{3}$	0.016	0.055
$\Omega _{4}$	0.079	0.085

**Table 14:** Ranking of D-SF-CoCoSo Method.

Alternatives	$\tau _{\imath }^{1}$	$\tau _{\imath }^{2}$	$\tau _{\imath }^{3}$	$\tau _{\imath }$	Ranking
$\Omega _{1}$	0.278	5.964	0.842	3.479	2
$\Omega _{2}$	0.250	4.340	0.756	2.718	3
$\Omega _{3}$	0.143	2.000	0.432	1.356	4
$\Omega _{4}$	0.329	6.389	0.995	3.850	1

### Comparative analysis with spherical fuzzy information

In this subsection, SF information from current literature is used. This data is translated into the D-SF structure, allowing us to compare the ranks obtained by our proposed approach to those provided by current aggregation processes in the literature.

The distinct benefits of our robust strategy is highlighted, distinguishing it from others. To provide a complete evaluation, the comparison of proposed method to logarithmic-based aggregation approaches^52^ and the Einstein AOs developed by Ashraf et al.^53^. This extensive evaluative contrast reveals how the proposed AOs and method outperforms in handling challenging everyday problems in DM scenarios, demonstrating exceptional efficacy and robustness.

An extensive method is employed to properly evaluate the proposed AOs and hybrid method. Table 15 shows an aggregated normalized SF dataset based on Ashraf’s et al. work^53^.

**Table 15:** SF Matrix for Comparison.

	$\gimel _{1}$	$\gimel _{2}$	$\gimel _{3}$	$\gimel _{4}$
$\Omega _{1}$	$(0.78,0.22,0.31)$	$(0.78,0.31,0.20)$	$(0.69,0.40,0.29)$	$(0.76,0.23,0.37)$
$\Omega _{2}$	$(0.80,0.07,0.27)$	$(0.67,0.24,0.34)$	$(0.81,0.16,0.22)$	$(0.91,0.18,0.09)$
$\Omega _{3}$	$(0.81,0.12,0.22)$	$(0.89,0.16,0.09)$	$(0.74,0.28,0.22)$	$(0.67,0.15,0.39)$
$\Omega _{4}$	$(0.70,0.26,0.27)$	$(0.53,0.32,0.39)$	$(0.61,0.42,0.43)$	$(0.57,0.25,0.42)$
$\Omega _{5}$	$(0.63,0.33,0.27)$	$(0.64,0.29,0.36)$	$(0.72,0.36,0.22)$	$(0.68,0.41,0.29)$

The data was transformed to the suggested D-SF structure (see to Table 16), allowing for different aggregate operations and proposed method on the dataset.

**Table 16:** D-SF decision matrix.

	$\gimel _{1}$	$\gimel _{2}$	$\gimel _{3}$	$\gimel _{4}$
$\Omega _{1}$	$(0.78,0.22,0.31;0.08)$	$(0.78,0.31,0.20;0.10)$	$(0.69,0.40,0.29;0.11)$	$(0.76,0.23,0.37;0.09)$
$\Omega _{2}$	$(0.80,0.07,0.27;0.10)$	$(0.67,0.24,0.34;0.17)$	$(0.81,0.16,0.22;0.03)$	$(0.91,0.18,0.09;0.19)$
$\Omega _{3}$	$(0.81,0.12,0.22;0.07)$	$(0.89,0.16,0.09;0.19)$	$(0.74,0.28,0.22;0.10)$	$(0.67,0.15,0.393;0.17)$
$\Omega _{4}$	$(0.70,0.26,0.27;0.15)$	$(0.53,0.32,0.39;0.07)$	$(0.61,0.42,0.43;0.10)$	$(0.57,0.25,0.42;0.08)$
$\Omega _{5}$	$(0.63,0.33,0.27;0.04)$	$(0.64,0.29,0.36;0.09)$	$(0.72,0.36,0.22;0.08)$	$(0.68,0.41,0.29;0.05)$

The converted SF information to D-SF information is subjected to the proposed aggregation operators and methodology. The resulting scoring outcomes are detailed in Table 17. Table 17 also summarizes the ranks of suggested Azcel-Alisna, logarithm-based, and Einstein AOs, as well as our proposed methodology. An detailed evaluation of these outcomes provides persuasive proof of our technique’s excellence and usefulness. Its ability to offer accurate solutions in complex DM settings demonstrates its potential to transform decision sciences and associated areas.

**Table 17:** Scoring and ranking.

	$\wp (\Omega _{1})$	$\wp (\Omega _{2})$	$\wp (\Omega _{3})$	$\wp (\Omega _{4})$	$\wp (\Omega _{5})$	Ranking
L-SFWA^52^	$0.982$	$0.998$	$0.984$	$0.737$	$0.934$	$\Omega _{2}>\Omega _{3}>\Omega _{1}>\Omega _{5}>\Omega _{4}$
L-SFOWA^52^	$0.980$	$0.993$	$0.987$	$0.613$	$0.903$	$\Omega _{2}>\Omega _{3}>\Omega _{1}>\Omega _{5}>\Omega _{4}$
L-SFHWA^52^	$0.9995$	$0.9999$	$0.9997$	$0.646$	$0.984$	$\Omega _{2}>\Omega _{3}>\Omega _{1}>\Omega _{5}>\Omega _{4}$
L-SFWG^52^	$0.979$	$0.995$	$0.972$	$0.622$	$0.926$	$\Omega _{2}>\Omega _{3}>\Omega _{1}>\Omega _{5}>\Omega _{4}$
L-SFOWG^52^	$0.976$	$0.979$	$0.973$	$0.330$	$0.892$	$\Omega _{2}>\Omega _{3}>\Omega _{1}>\Omega _{5}>\Omega _{4}$
L-SFHWG^52^	$0.9998$	$0.9999$	$0.9991$	$0.822$	$0.998$	$\Omega _{2}>\Omega _{3}>\Omega _{1}>\Omega _{5}>\Omega _{4}$
SFEWA^53^	$0.472$	$0.529$	$0.523$	$0.426$	$0.458$	$\Omega _{2}>\Omega _{3}>\Omega _{1}>\Omega _{5}>\Omega _{4}$
SFEWG^53^	$0.553$	$0.609$	$0.593$	$0.477$	$0.510$	$\Omega _{2}>\Omega _{3}>\Omega _{1}>\Omega _{5}>\Omega _{4}$
GSFEWA^53^	$0.512$	$0.561$	$0.555$	$0.470$	$0.495$	$\Omega _{2}>\Omega _{3}>\Omega _{1}>\Omega _{5}>\Omega _{4}$
GSFEWG^53^	$0.516$	$0.566$	$0.549$	$0.433$	$0.473$	$\Omega _{2}>\Omega _{3}>\Omega _{1}>\Omega _{5}>\Omega _{4}$
GSFEOWA^53^	$0.512$	$0.562$	$0.556$	$0.470$	$0.496$	$\Omega _{2}>\Omega _{3}>\Omega _{1}>\Omega _{5}>\Omega _{4}$
GSFEOWG^53^	$0.516$	$0.567$	$0.549$	$0.433$	$0.474$	$\Omega _{2}>\Omega _{3}>\Omega _{1}>\Omega _{5}>\Omega _{4}$
DSFAAWA	$0.149$	$0.262$	$0.203$	$0.095$	$0.096$	$\Omega _{2}>\Omega _{3}>\Omega _{1}>\Omega _{5}>\Omega _{4}$
DSFAAWG	$0.147$	$0.246$	$0.189$	$0.090$	$0.094$	$\Omega _{2}>\Omega _{3}>\Omega _{1}>\Omega _{5}>\Omega _{4}$
Proposed method	$0.471$	$0.810$	$0.631$	$0.300$	$0.318$	$\Omega _{2}>\Omega _{3}>\Omega _{1}>\Omega _{5}>\Omega _{4}$

### Discussion

This section discusses the comparison results derived from our analysis.

A comparative examination of our suggested technique is presented above, demonstrating its validity, reliability, and efficacy. This comparative research is divided into two components. First, we present a methodology for comparing our methods. Leveraging a numerical example solved using our suggested technique serves as the foundation for this comparison, aligning the ranks accordingly. Subsequently, we use SF information from current literature. This data is converted into our D-SF structure, allowing us to compare the rankings provided by our proposed technique to those obtained from current aggregation processes in the literature.

**Figure 4:**
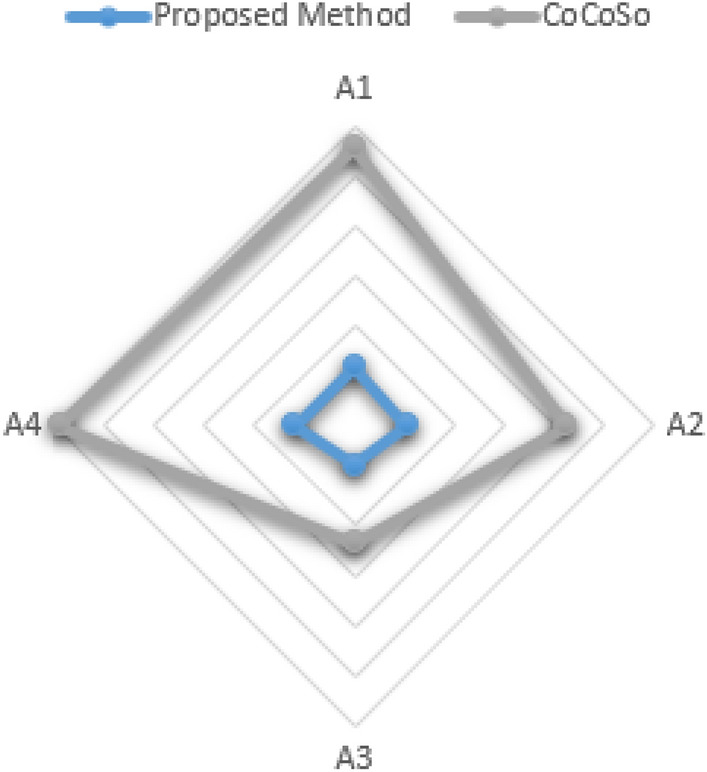
Comparison with CoCoSo.

For the first portion of our comparison, the alignment of ranks between our suggested technique and the CoCoSo method demonstrates the convergence of solutions, emphasizing our methodology’s robustness and efficacy. The constant results show the legitimacy and trustworthiness of our technique in the context of D-SF. This agreement indicates that our methodology routinely produces findings similar to those of the well-known CoCoSo technique, increasing our trust in its dependability. Such consistency instills confidence in our suggested method’s applicability and practicality for DM in the D-SF domain. The accompanying graph (see Figure 4) provides a clear and intelligible comparison between our proposed technique with the CoCoSo method. The findings shown are simply interpretable, which strengthens the validity of our technique.

**Figure 5:**
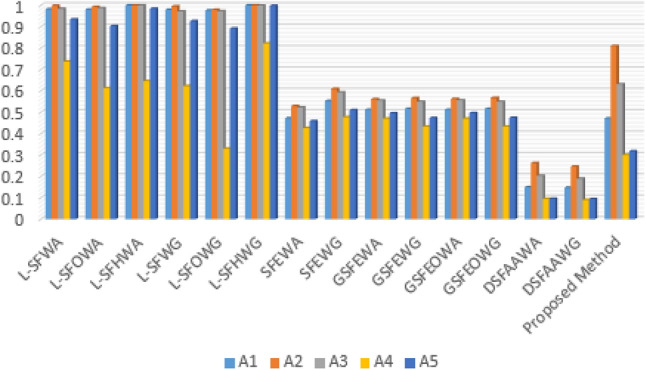
Comparative score ranking.

Comparing SFS-converted DSF data presents a similar ranking result. The alignment of ranks between our suggested methodology and Aczel–Alsina AOs and the logarithmic and Einstein based^52,53^ AOs approach enhances the technique’s robustness and trustworthiness inside the D-SF framework. The graphical depiction in Figure 5 of this comparison improves the clarity and persuasiveness of our findings, increasing confidence in the validity and usefulness of our suggested technique. Figure 5 illustrates how the score results are presented graphically.

## Conclusion

To sum up, this paper has established the groundwork for an innovative way of DM for crude oil pretreatment. As an extension of Spherical Fuzzy Sets, we have explored the unique notion of Disc Spherical Fuzzy Sets (D-SFSs) and shown its potential within the Aczel Alsina norm. The basic workings of the Aczel Alsina norm in D-SFSs were outlined and supported, offering a thorough comprehension of this novel paradigm. Primarily, the study pioneered the field by introducing, proving, and presenting aggregation procedures particular to Aczel Alsina norm in D-SFSs. Through a convincing case study on crude oil preprocessing, the suggested MAGDM algorithm, MEREC-SAWAR-MARCOS-D-SFSs, integrates these aggregation operations and showcases its use. A thorough comparison with CoCoSo was used to prove the reliability of our methodology. In essence, this research not only expands the theoretical landscape of DM but also offers a practical and applicable framework, particularly relevant in the intricate domain of crude oil pretreatment. In the trajectory of our future work, we plan to include more norms in the aggregation operations of D-SFSs. In addition, we are going to explore how different MADM approaches might be integrated in the context of D-SFSs in our future study. We aim to demonstrate the superiority, robustness, and competence of D-SFSs to handle difficult decision scenarios by utilizing a variety of MADM techniques. This exploration is poised to contribute valuable insights into the broader understanding of decision science and reinforce the position of D-SFSs as a formidable tool in MADM.

The D-SF aggregation operator enhances DM by effectively handling complex data and uncertainties, leading to improved decision quality and consistency. Its flexibility and adaptability allow for comprehensive analysis and integration with other operators, ensuring robust and reliable outcomes in diverse decision contexts.

## Data Availability

No data were used in this study.

## References

[1] Thompson, D. G., Taylor, A. S. & Graham, D. E. Emulsification and demulsification related to crude oil production. *Colloids Surf.* **15**, 175–189 (1985).

[2] Ye, G. *et al.* Application of ultrasound on crude oil pretreatment. *Chem. Eng. Process.* **47**(12), 2346–2350 (2008).

[3] Guoxiang, Y., Xiaoping, L., Fei, P., Pingfang, H. & Xuan, S. H. E. N. Pretreatment of crude oil by ultrasonic-electric united desalting and dewatering. *Chin. J. Chem. Eng.* **16**(4), 564–569 (2008).

[4] Hu, Y., Gong, M., Feng, S., Xu, C. C. & Bassi, A. A review of recent developments of pre-treatment technologies and hydrothermal liquefaction of microalgae for bio-crude oil production. *Renew. Sustain. Energy Rev.* **101**, 476–492 (2019).

[5] Khajehesamedini, A., Sadatshojaie, A., Parvasi, P., Rahimpour, M. R. & Naserimojarad, M. M. Experimental and theoretical study of crude oil pretreatment using low-frequency ultrasonic waves. *Ultrason. Sonochem.* **48**, 383–395 (2018). 30080563 10.1016/j.ultsonch.2018.05.032

[6] Babalola, F. U. & Susu, A. A. Pre-treatment of heavy crude oils for refining. In *Processing of Heavy Crude Oils-Challenges and Opportunities*. IntechOpen.

[7] Hajeeh, M. A. Water desalination plants performance using fuzzy multi-criteria decision making. *WSEAS Trans. Syst.* **9**(4), 422–431 (2010).

[8] Fetanat, A. & Tayebi, M. A picture fuzzy set-based decision support system for treatment technologies prioritization of petroleum refinery effluents: A circular water economy transition towards oil & gas industry. *Sep. Purif. Technol.* **303**, 122220 (2022).

[9] Wu, W., Huang, P. & Geng, S. Application of interval-valued Pythagorean fuzzy VIKOR approach for petroleum sludge treatment technology evaluation and selection. *Environ. Sci. Pollut. Res.* **28**(36), 50890–50907 (2021). 10.1007/s11356-021-14225-6 33973115

[10] Fetanat, A. & Tayebi, M. Sustainability and resilience-oriented prioritization of oil and gas produced water treatment technologies: A novel decision support system under circular intuitionistic fuzzy set. *Geoenergy Sci. Eng.* **221**, 211379 (2023).

[11] Zadeh, L. A. Fuzzy sets. *Inf. Control* **8**(3), 338–353 (1965).

[12] Kahraman, C., Onar, S. C. & Oztaysi, B. Fuzzy multicriteria decision-making: A literature review. *Int. J. Comput. Intell. Syst.* **8**(4), 637–666 (2015).

[13] Atanassov, K. T. & Stoeva, S. Intuitionistic fuzzy sets. In *Fuzzy Sets and Systems* **20**(1), 87–96 (1986)

[14] Atanassov, K. T. Circular intuitionistic fuzzy sets. *J. Intell. Fuzzy Syst.* **39**(5), 5981–5986 (2020).

[15] Atanassov, K. & Marinov, E. Four distances for circular intuitionistic fuzzy sets. *Mathematics* **9**(10), 1121 (2021).

[16] Kahraman, C. & Alkan, N. Circular intuitionistic fuzzy TOPSIS method with vague membership functions: Supplier selection application context. *Notes Intuit. Fuzzy Sets* **27**(1), 24–52 (2021).

[17] Ashraf, S., Chohan, M. S., Muhammad, S. & Khan, F. Circular intuitionistic fuzzy TODIM approach for material selection for cryogenic storage tank for liquid nitrogen transportation. *IEEE Access*10.1109/ACCESS.2023.3312568 (2023).

[18] Garg, H., Ünver, M., Olgun, M. & Trkarslan, E. An extended EDAS method with circular intuitionistic fuzzy value features and its application to multi-criteria decision-making process. *Artif. Intell. Rev.* **56**, 1–32 (2023).

[19] Yager, R. R. Pythagorean membership grades in multicriteria decision making. *IEEE Trans. Fuzzy Syst.* **22**(4), 958–965 (2013).

[20] Garg, H. A new generalized Pythagorean fuzzy information aggregation using Einstein operations and its application to decision making. *Int. J. Intell. Syst.* **31**(9), 886–920 (2016).

[21] Liang, D. & Xu, Z. The new extension of TOPSIS method for multiple criteria decision making with hesitant Pythagorean fuzzy sets. *Appl. Soft Comput.* **60**, 167–179 (2017).

[22] Bozyigit, M. C., Olgun, M. & Ünver, M. *Circular Pythagorean Fuzzy Sets and Applications to Multi-criteria Decision Making*. arXiv preprint arXiv:2210.15483 (2022).

[23] Khan, M. J., Alcantud, J. C. R., Kumam, W., Kumam, P. & Alreshidi, N. A. Expanding Pythagorean fuzzy sets with distinctive radii: Disc Pythagorean fuzzy sets. *Complex Intell. Syst.* **9**, 1–18 (2023).

[24] Olgun, M. & Ünver, M. Circular Pythagorean fuzzy sets and applications to multi-criteria decision making. *Informatica* **34**(4), 713–742 (2023).

[25] Alsattar, H. A. *et al.* Three-way decision-based conditional probabilities by opinion scores and Bayesian rules in circular-Pythagorean fuzzy sets for developing sustainable smart living framework. *Inf. Sci.* **649**, 119681 (2023).

[26] Ashraf, S., Abdullah, S., Mahmood, T., Ghani, F. & Mahmood, T. Spherical fuzzy sets and their applications in multi-attribute decision making problems. *J. Intell. Fuzzy Syst.* **36**(3), 2829–2844 (2019).

[27] Ashraf, S. & Abdullah, S. Spherical aggregation operators and their application in multiattribute group decision-making. *Int. J. Intell. Syst.* **34**(3), 493–523 (2019).

[28] Rafiq, M., Ashraf, S., Abdullah, S., Mahmood, T. & Muhammad, S. The cosine similarity measures of spherical fuzzy sets and their applications in decision making. *J. Intell. Fuzzy Syst.* **36**(6), 6059–6073 (2019).

[29] Khan, M. J., Kumam, P., Deebani, W., Kumam, W. & Shah, Z. Distance and similarity measures for spherical fuzzy sets and their applications in selecting mega projects. *Mathematics* **8**(4), 519 (2020).

[30] Ashraf, S., Chohan, M. S., Ahmad, S., Hameed, M. S. & Khan, F. Decision aid algorithm for kidney transplants under disc spherical fuzzy sets with distinctive radii information. *IEEE Access*10.1109/ACCESS.2023.3327830 (2023).

[31] Ashraf, S., Iqbal, W., Ahmad, S. & Khan, F. Circular spherical fuzzy Sugeno weber aggregation operators: A novel uncertain approach for adaption a programming language for social media platform. *IEEE Access*10.1109/ACCESS.2023.3329242 (2023).

[32] Keshavarz-Ghorabaee, M., Amiri, M., Zavadskas, E. K., Turskis, Z. & Antucheviciene, J. Determination of objective weights using a new method based on the removal effects of criteria (MEREC). *Symmetry* **13**(4), 525 (2021).

[33] Marinkovic, M. *et al.* Application of wasted and recycled materials for production of stabilized layers of road structures. *Buildings* **12**(5), 552 (2022).

[34] Mishra, A. R. *et al.* An integrated decision support framework using single-valued-MEREC-MULTIMOORA for low carbon tourism strategy assessment. *IEEE Access* **10**, 24411–24432 (2022).

[35] Wan, G., Rong, Y. & Garg, H. An efficient spherical fuzzy MEREC-CoCoSo approach based on novel score function and aggregation operators for group decision making. *Granul. Comput.* **8**, 1–23 (2023). 10.1007/s41066-023-00381-2 PMC10184975 38625159

[36] Gao, K. *et al.* An integrated spherical fuzzy multi-criterion group decision-making approach and its application in digital marketing technology assessment. *Int. J. Comput. Intell. Syst.* **16**(1), 125 (2023).

[37] Keruliene, V., Zavadskas, E. K. & Turskis, Z. Selection of rational dispute resolution method by applying new step-wise weight assessment ratio analysis (SWARA). *J. Bus. Econ. Manag.* **11**(2), 243–258 (2010).

[38] Chen, T. Y. Multiple criteria decision analysis under complex uncertainty: A Pearson-like correlation-based Pythagorean fuzzy compromise approach. *Int. J. Intell. Syst.* **34**(1), 114–151 (2019).

[39] Tas, M. A., Çakir, E. & Ulukan, Z. Spherical fuzzy SWARA-MARCOS approach for green supplier selection. *Tecnologia* **3C**, 115–133 (2021).

[40] Chaurasiya, R. & Jain, D. A new algorithm on Pythagorean fuzzy-based multi-criteria decision-making and its application. *Iran. J. Sci. Technol. Trans. Electr. Eng.* **47**, 1–16 (2023).

[41] Rani, P., Mishra, A. R., Krishankumar, R., Ravichandran, K. S. & Gandomi, A. H. A new Pythagorean fuzzy based decision framework for assessing healthcare waste treatment. *IEEE Trans. Eng. Manage.* **69**(6), 2915–2929 (2020).

[42] Rani, P. *et al.* Pythagorean fuzzy SWARA-VIKOR framework for performance evaluation of solar panel selection. *Sustainability* **12**(10), 4278 (2020).

[43] Stevic, Ž, Pamucar, D., Puška, A. & Chatterjee, P. Sustainable supplier selection in healthcare industries using a new MCDM method: Measurement of alternatives and ranking according to COmpromise solution (MARCOS). *Comput. Ind. Eng.* **140**, 106231 (2020).

[44] Badi, I. & Pamucar, D. Supplier selection for steelmaking company by using combined Grey-MARCOS methods. *Decis. Mak. Appl. Manag. Eng.* **3**(2), 37–48 (2020).

[45] Stankovic, M., Stevic, Ž, Das, D. K., Subotic, M. & Pamucar, D. A new fuzzy MARCOS method for road traffic risk analysis. *Mathematics* **8**(3), 457 (2020).

[46] Puška, A., Stojanovic, I., Maksimovic, A. & Osmanovic, N. Evaluation software of project management by using measurement of alternatives and ranking according to compromise solution (MARCOS) method. *Op. Res. Eng. Sci. Theory Appl.* **3**(1), 89–102 (2020).

[47] Chaurasiya, R. & Jain, D. Generalized intuitionistic fuzzy entropy on IF-MARCOS technique in multi-criteria decision making. In *Advances in Computing and Data Sciences: 5th International Conference, ICACDS 2021, Nashik, India, April 23–24, 2021, Revised Selected Papers, Part I 5* 592-603 (Springer International Publishing, 2021).

[48] Kumar, R. *et al.* A multi-perspective benchmarking framework for estimating usable-security of hospital management system software based on fuzzy logic, ANP and TOPSIS methods. *KSII Trans. Internet Inf. Syst. (TIIS)* **15**(1), 240–263 (2021).

[49] Aczél, J. & Alsina, C. Characterizations of some classes of quasilinear functions with applications to triangular norms and to synthesizing judgements. *Aequ. Math.* **25**(1), 313–315 (1982).

[50] Ali, J. Spherical fuzzy symmetric point criterion-based approach using Aczel–Alsina prioritization: Application to sustainable supplier selection. *Granul. Comput.* **9**(2), 33 (2024).

[51] Ali, J. Analysis and application of r, s, t-spherical fuzzy Aczel–Alsina aggregation operators in multiple criteria decision-making. *Granul. Comput.* **9**(1), 17 (2024).

[52] Jin, Y., Ashraf, S. & Abdullah, S. Spherical fuzzy logarithmic aggregation operators based on entropy and their application in decision support systems. *Entropy* **21**(7), 628 (2019). 33267343 10.3390/e21070628 PMC7515119

[53] Ashraf, S., Abdullah, S. & Chinram, R. Emergency decision support modeling under generalized spherical fuzzy Einstein aggregation information. *J. Ambient Intell. Humaniz. Comput.* **13**, 1–27 (2022). 10.1007/s12652-021-03493-2 PMC8475448 34603537

