# Author Correction: Enhanced decision technique for optimized crude oil pretreatment under disc spherical fuzzy Aczel Alsina aggregation information

**DOI:** 10.1038/s41598-024-69328-0

**Published:** 2024-08-09

**Authors:** Qazi Adnan Ahmad, Shahzaib Ashraf, Wania Iqbal, Ma Li Qiang

**Affiliations:** 1 School of Mines, China University of Mining and Technology, Xuzhou, Jiangsu China; 2 Institute of Mathematics, Khwaja Fareed University of Engineering & Information Technology, Rahim Yar Khan, 64200 Pakistan; 3 College of Energy and Mining Engineering, Shandong University of Science and Technology, Qingdao, 266590 China; 4 Key Laboratory of Xinjiang Coal Resources Green Mining (Xinjiang Institute of Engineering), Ministry of Education, Ürümqi, 830023 China

Correction to: *Scientific Reports* 10.1038/s41598-024-62036-9, published online 02 July 2024

The Acknowledgements section in the original version of this Article was incomplete.

“This paper was supported by National Natural Science Foundation of China (Grant No. 42250410333).”

now reads:

“This paper was fully supported by National Natural Science Foundation of China (Grant No. 42250410333), and also by Natural Science Foundation of China (NSFC), (Grant No. 51874280) and Fundamental Research Funds for the Central Universities (2021ZDPY0211); Research and Engineering Demonstration of Low Cost Large Scale Purification and Cascade Utilization Technology for Mining Brackish Water in the Zhundong Region (2023B03009).”

The original Article has been corrected.

